# Green Synthesis, Characterization and Application of Silver Nanoparticles Using Bioflocculant: A Review

**DOI:** 10.3390/bioengineering11050492

**Published:** 2024-05-15

**Authors:** Nkanyiso C. Nkosi, Albertus K. Basson, Zuzingcebo G. Ntombela, Nkosinathi G. Dlamini, Rajasekhar V. S. R. Pullabhotla

**Affiliations:** 1 Biochemistry and Microbiology Department, Faculty of Science, Agriculture, and Engineering, P/Bag X1001, University of Zululand, KwaDlangezwa 3886, South Africa; bassona@unizulu.ac.za (A.K.B.); ntombelaz@unizulu.ac.za (Z.G.N.); dlamining@unizulu.ac.za (N.G.D.); 2 Chemistry Department, Faculty of Science, Agriculture, and Engineering, P/Bag X1001, University of Zululand, KwaDlangezwa 3886, South Africa

**Keywords:** bioflocculant, nanoparticles, biosynthesis, characterization, potential application

## Abstract

Nanotechnology has emerged as an effective means of removing contaminants from water. Traditional techniques for producing nanoparticles, such as physical methods (condensation and evaporation) and chemical methods (oxidation and reduction), have demonstrated high efficiency. However, these methods come with certain drawbacks, including the significant energy requirement and the use of costly and hazardous chemicals that may cause nanoparticles to adhere to surfaces. To address these limitations, researchers are actively developing alternative procedures that are cost-effective, environmentally safe, and user-friendly. One promising approach involves biological synthesis, which utilizes plants or microorganisms as reducing and capping agents. This review discusses various methods of nanoparticle synthesis, with a focus on biological synthesis using naturally occurring bioflocculants from microorganisms. Bioflocculants offer several advantages, including harmlessness, biodegradability, and minimal secondary pollution. Furthermore, the review covers the characterization of synthesized nanoparticles, their antimicrobial activity, and cytotoxicity. Additionally, it explores the utilization of these NPs in water purification and dye removal processes.

## 1. Introduction

In the modern era, issues with drinking water are common in most countries and are particularly severe in developing nations [1]. The world faces significant challenges in meeting the increasing demand for clean water due to factors such as prolonged droughts, rapid population growth, stricter health regulations, and rising demands [2]. Clean water, devoid of toxic chemicals and pathogens, is crucial for human health [2]. Protecting water treatment systems against potential chemical and biological threats is essential for effective water resource management [3]. Currently, various techniques for water treatment are employed, including chemical and physical agents such as chlorine and its derivatives. However, the use of commercial chemicals poses risks to human health and the environment [4]. Alternatively, bioflocculants which are substances formed by microbes, like yeast, fungi, bacterium, and algae (as they grow) have been seen as a promising substitute method for wastewater treatment because of their biodegradability, lack of secondary pollution, and harmlessness to nature and aquatic life forms [5]. However, bioflocculants have limitations, such as low yield, short shelf-life, and low flocculating activity, which restrict their industrial applications [6]. Therefore, there is a pressing need to explore new eco-friendly technologies for wastewater purification.

Over the past decade, nanotechnology has been seen to possess the ability to revolutionize the field of wastewater treatment [7]. It involves the use of nanoscale materials and devices to enhance the treatment process efficiency, reduce energy consumption, and enhance the quality of treated water [8]. Nanotechnology is a field of science and engineering that deals with the design, production, and application of materials, structures, devices, and systems with dimensions ranging from 1 to 100 nanometers (nm) [9,10,11]. It has the potential to change many aspects of our existence, including medicine, electronics, energy, and materials science [9,12]. Nanomaterials can be customized to target specific cells or tissues in the body, which allows for more precise drug delivery [13]. They can also be utilized for imaging, such as in magnetic resonance imaging (MRI), and for sensing and detecting diseases at an early stage [14,15]. However, there are also concerns about the potential risks and unintended consequences of nanotechnology, such as the toxicity of some nanoparticles and the potential for environmental harm [16]. Therefore, careful regulation and evaluation of the safety of nanomaterials and devices is important as the field continues to develop [17].

Various synthesis technologies for nanoparticles, encompassing both physical and chemical methods, have been extensively documented [18]. Nonetheless, it has become apparent that certain procedures possess inherent drawbacks, notably the substantial energy requirements and the use of harmful chemicals [19]. In response to these challenges, recent years have witnessed a notable shift in nanotechnology research toward the development of environmentally sustainable and economically viable synthesis methods for nanoparticles. This strategic pivot aims to address the escalating demand for nanoparticles across diverse industries [8,19]. One promising approach involves the utilization of extracts derived from plants or microbes as an alternative to conventional chemical and physical techniques, which often rely on toxic substances and produce hazardous waste [20]. An inherent advantage of this ‘green’ synthesis approach lies in its ability to yield nanoparticles with tailored properties and functions, such as catalytic, adsorption, and antibacterial activities, contingent upon the specific type of plant extract or microorganism employed [21]. Furthermore, nanoparticles synthesized through green methods typically exhibit biocompatibility and biodegradability, rendering them suitable for a wide array of environmental applications, including wastewater treatment [22].

Among other metals, silver NPs have shown immense promise in various uses and have contributed positively to the development of nanotechnology [23]. Silver nanostructures have gained prominence over the past two decades due to their distinct features, which differ from bulk silver. These properties include the ability to kill microbes, electrical conductivity, thermal conductivity, optical properties, and mechanical strength [24]. The antimicrobial properties of silver nanoparticles (AgNPs) have made them particularly useful in the fields of medicine and therapy, as they can potentially replace traditional antibiotics, which are becoming less effective due to increasing bacterial resistance [25]. Additionally, developing new antibiotics is expensive and time-consuming, making AgNPs a more attractive option [26]. AgNPs can be designed to avoid toxicity in cells, which is important for their medical use [27]. Due to their small size, they can accumulate on the surface of bacteria and penetrate their membranes, leading to their death.

Another useful application of AgNPs is their ability to coat surfaces and prevent bacterial growth, making them valuable in the development of sterile catheters capable of preventing venous and urinary tract infections. Green-synthesized nanoparticles are being investigated for potential use in wastewater treatment. Some of the nanoparticles under investigation include silver, copper, iron, and zinc oxide nanoparticles [10,28,29]. These nanoparticles can remove pollutants such as heavy metals, organic compounds, and dyes, from wastewater through various mechanisms, including adsorption, oxidation, and reduction [30]. Numerous studies have synthesized nanoparticles using plants, including *Brassica oleracea* var. botrytis [31], *Ephedra Alata* [32], *Berberis vulgaris* [33], *Fagonia cretica* [34], *Andrographis alata* [35], *Cymbopogon citratus* [36], *Sumac* [37], *Momordica charantia* and *Curcuma zedoaria* [38], *Bombax ceiba* [39], *Aegle marmelos* [40]. However, only a few studies have explored the synthesis of nanoparticles using bioflocculants. For instance, Dlamini et al. [41], Adebayo-Tayo et al. [42], Ntombela et al. [10], and Tsilo et al. [43] synthesized copper, silver, zinc oxide, and iron nanoparticles using bioflocculants.

As a result, the objective of this study is to close the knowledge gap by examining the most recent improvements in AgNP synthesis utilizing microbial bioflocculants from January 2015 to December 2023. Furthermore, the research examines the characterization and use of biosynthesized AgNPs in wastewater treatment [22].

## 2. Procedures for the Production of Silver NPs

Various techniques can be employed to prepare AgNPs, and these can be categorized as top-down, bottom-up, chemical, and biological procedures.

### 2.1. Top-Down and Bottom-Up Approaches

**Figure 1:**
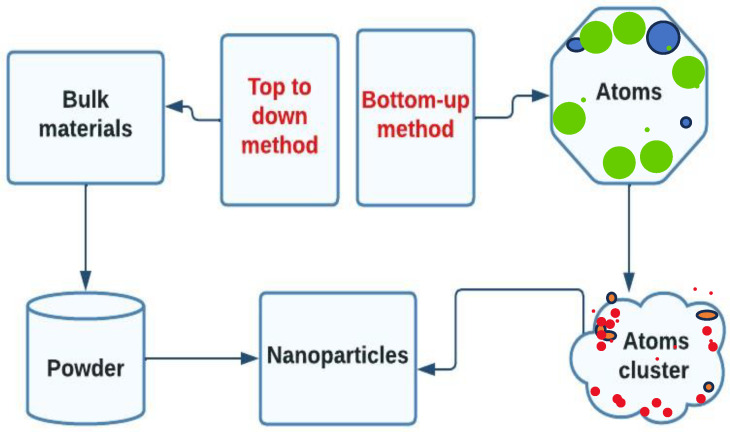
Top-down and bottom-up methods to synthesize NPs [56]. Created with Lucidchart.

**Figure 2:**
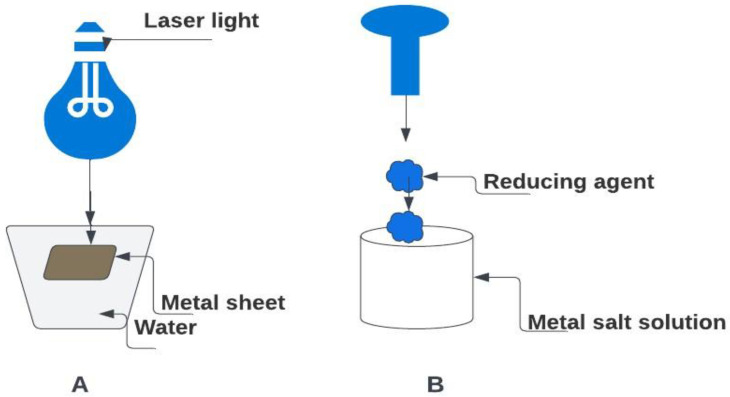
Physical method (**A**) and chemical method (**B**) [56]. Created with Lucidchart.

This section explores a diversity of methods for synthesizing silver nanoparticles encompassing top-down, bottom-up, chemical, and physical approaches [44]. The top-down method involves employing massive components as initial materials and utilizing physical processes to break down large particles into smaller NPs, as illustrated in Figure 1 [45]. Physical methods often utilized in this approach include mechanical milling, laser ablation, and sputtering [46]. In mechanical milling, starting materials in powder form are loaded into a milling machine along with milling balls made of materials such as steel, ceramic, or glass. The milling machine generates high impact and frictional force between the milling balls and the starting materials, resulting in the production of nanoparticles [47]. Laser ablation employs a high-power laser to vaporize a target material in a liquid medium, producing a plasma plume consisting of high-energy particles and atoms. These particles and atoms rapidly cool down and condense into nanoparticles within the liquid medium. By adjusting laser parameters such as pulse duration, intensity, and wavelength, along with the characteristics of the target material and the liquid medium, it is possible to control the size and shape of the resulting NPs, as demonstrated in Figure 2A [48].

The sputtering technique encompasses the production of NPs through the physical vapor deposition (PVD) method. To carry out the sputtering process, high-energy particles, usually ions, are directed at a solid surface called the target within a vacuum chamber, causing atoms or ions to be ejected from it. The ejected particles can subsequently settle on a substrate, forming thin films or, under specific circumstances, forming nanoparticles [49]. However, this method may not be appropriate for producing precise metallic nanoparticles, particularly when a small distribution size is required.

The bottom-up technique in nanoparticle synthesis involves assembling nanoparticles from molecular components, contrasting with the top-down method that starts with bulk materials and breaks them down into smaller nanoparticles [50]. In the case of silver nanoparticle formation using the bottom-up approach, silver salts like silver nitrate (AgNO_3_) are chemically reduced with agents such as sodium borohydride, hydrazine, ascorbic acid, or trisodium citrate, leading to controlled nucleation and development of silver nanoparticles with varying sizes and shapes (Figure 2B) [51]. Methods like electrochemical reduction, microemulsion techniques, and photochemical procedures exemplify bottom-up strategies, enabling the in situ production of well-dispersed nanoparticles under mild reaction conditions [52]. While bottom-up techniques are known for creating nanoparticles with a limited size distribution compared to top-down methods like laser ablation or sputtering, they may suffer from poor mixing causing blockages, scalability challenges for high-volume production, and issues with process reliability and reproducibility [53]. To address these drawbacks and promote eco-friendliness in nanoparticle synthesis, there is a growing imperative to develop more sustainable and environmentally friendly methods [54]. By exploring alternative approaches such as biological synthesis using microorganisms, plant extracts, or enzymes, researchers can mitigate the environmental impact of nanoparticle production and enhance the overall sustainability of nanotechnology. These eco-friendly methods offer advantages in terms of reduced toxicity, cost-effectiveness, controlled synthesis parameters, and scalability, making them promising avenues for the future of nanoparticle synthesis [55].

### 2.2. Biological Methods for Synthesizing Nanoparticles

Though chemical methods are a simple way to produce Ag nanoparticles, the use of toxic and expensive reducing agents limits their industrial applications [57]. Toxic reagents have the potential to generate problems when it comes to biological downstream uses along with environmental issues. As a solution to this issue, an eco-friendly technique for synthesizing AgNPs that eliminates the use of harmful chemicals is required [58]. The biological synthesis method is gaining more consideration because it is considered a sustainable, eco-friendly, cost-effective, and non-hazardous technique [59]. The biological procedures for synthesizing AgNPs involve the use of microorganisms or their by-products, as well as plants or plant extracts [60]. Carbohydrates, lipids, nucleic acids, and proteins are the biological components used in the biosynthesis processes, which offer advantages such as being widely available, inexpensive, easy to synthesize, and environmentally friendly [61]. The AgNPs synthesized through green synthesis have many biological uses including as: microbicidal, anticancer, antiphlogistic, antioxidant, drug-carrier, wastewater-treatment, and dye-removal agents [62]. The synthesis of silver NPs utilizing living things including fungi, algae, bacteria, and plants has been described [59]. These methods are discussed in detail below.

#### 2.2.1. Green Synthesis of Silver Nanoparticles Using Plant Extracts

Biosynthesis is a biosynthetic mechanism that produces AgNPs by utilizing plant resources as stabilizing substances. This approach has recently become popular due to its numerous benefits, including: low cost, accessibility, synthesis at room temperature, environmentally friendliness, and non-toxicity [63]. Plant extracts are rich in many types of bioactive compounds, including flavonoids, tannins, and enzymes, which make the synthesis of bioactive AgNPs more stable and straightforward [64].

The primary approach for extracting plant materials for nanoparticle production involves washing and boiling plant materials, such as leaves, fruits, or roots, in water [65]. This extraction technique is based on isotropic distillation [66]. The extracted plant material is then filtered, usually with a muslin cloth or Whatman filter paper, to produce the final extract [67]. In addition to water-based extraction, other solvents such as ethanol have also been utilized to extract plant components for nanoparticle production [68]. The filtered plant extract is then used as a reducing and stabilizing agent, facilitating the green production of metal nanoparticles. The plant extract is combined with a metal precursor solution (for example, silver nitrate or gold chloride) under different reaction parameters such as pH, temperature, and time. Then, a centrifuge is utilized to separate the nanoparticles from the plant extract.

The plant extract contains phytochemicals such as flavonoids, terpenoids, sugars, and other biomolecules, which decrease metal ions and stabilize nanoparticle production [69]. The kind and concentration of phytochemicals, metal salt concentration, pH, and temperature all influence the rate of nanoparticle production, as well as their size, shape, and stability [70]. The plant extract serves a dual purpose, functioning as both a reducing and stabilizing agent, allowing for the one-pot green production of nanoparticles [71]. The search results show that this green, plant-based technique is a simpler, more convenient, cost-effective, and environmentally friendly way for nanoparticle production than traditional chemical approaches [72].

Phull et al. [73] indicated the technique for synthesizing metallic nanoparticles using *Euphorbia wallichii* extract. The researchers use a living plant system for producing silver nanoparticles. Silver may be absorbed by *Euphorbia wallichii* roots and transferred to plant shoots in the same oxidation state using an agar media. The Ag atoms rearranged themselves in the shoots to generate silver nanoparticles [74]. Jini and Sharmila [75] utilized a green production procedure with the help of an aqueous extract of *Allium cepa* as a stabilizing agent to produce silver NPs. The color of the mixture changed from transparent to brown, signifying the successful production of silver nanoparticles.

Hekmati and their co-workers employed a green procedure to synthesize silver NPs. They combined an extract obtained from three plant species, namely *Allium rotundum*, *Falcaria vulgaris Bernh*, and *Ferulago angulate Boiss*, with a solution of 0.01 M silver nitrate. After letting the mixture sediment at room temperature for a day, they observed a color change and used a UV–visible spectrophotometer to obtain absorbance, which confirmed the formation of Ag nanoparticles [76].

Castor oil, which is derived from *Ricinus communis* L. plant seeds, is a thick fluid that is commonly used to treat inflammation. Researchers utilized ricinoleic acid (RA) and epoxide ricinoleic acid (ERA) as agents to reduce and stabilize silver nanoparticles that were synthesized in an alkaline environment. The researchers found that the silver nanoparticles that were capped with RA were effective in detecting cysteine and that those prepared with RA were highly stable due to the lengthy preparation time. To obtain RA, castor oil was subjected to alkaline hydrolysis and then epoxidation to form ERA. The resulting surfactants were then precipitated using sodium hydroxide. The concentration of both Ag and surfactants was varied to optimize the yield of silver nanoparticles [77].

Another investigation reported by Singh and colleagues utilized an aqueous extract obtained from *Couroupita guianensis Aubl* flower to synthesize AgNPs and determined its capacity to collect free radicals using a 2,2-diphenyl-1-picrylhydrazyl (DPPH) assay [78].

Another work by Verma and Preet [79] utilized an extract from the *Jatropha integerrima* flower to synthesize AgNPs, which were then tested for anti-dengue activity through cytotoxicity assessment. The test was carried out at an ambient temperature and observed within 24 h to detect any change in the color of the mixture from colorless to dark brown, which would show the production of silver NPs. The silver NPs were also evaluated against the aquatic larval stages of *Aedes aegypti*, with the most significant mortality rates being observed when the AgNPs were combined with the plant’s leaf and flower extract. Nahar et al. [80] described the eco-friendly production of silver NPs utilizing leaf extract of *Clerodendrum viscosum*. The production of silver NPs was verified by a change in color from colorless to light yellow after mixing *Clerodendrum viscosum* leaf extract with 0.01 M silver NO_3_ solution. The color then changed from yellow light to brownish and then became brown-dark after 60 min at 25 °C. The color intensified with incubation time, and the yellowish solvent turned brown and dark after 60 min due to a greater amount of NP concentration. There was no more color change after 24 h. The silver NP color brown was due to SPR in the media. Huq et al. [81] stated that plant extracts contain various components that perform crucial functions in silver salt reduction and act as capping and stabilizing agents.

#### 2.2.2. Green Synthesis of Silver Nanoparticles Using Fungi

The utilization of fungi to produce silver NPs has garnered considerable curiosity as a result of its financial advantages and wide-ranging promise in various disciplines. Fungi, which encompass a diverse group of organisms, possess a variety of enzymes and proteins with the potential to diminish and stabilize metal nanoparticles [82]. Extensive research has been done on the production of silver NPs using fungi, and more than 120 fungal species from various taxa, such as Ascomycota (*Alternaria*), were discovered to be capable of forming nanosilver [83]: *Helvella* [84], *Fomes* [85], *Fomitopsis* [86], *Penicillium* [87], *Picoa* [88], *Penicillium oxalicum* [89], and *Sclerotium rolfsii* [90].

Basidiomycetes have attracted considerable attention as potential candidates for creating nanoparticles [91]. Scientists have directed their attention toward the examination of edible and medical mushrooms, which are frequently grown under controlled conditions. These particular fungi yield multiple biological compounds that can serve as stabilizers and capping agents. Furthermore, these compounds possess numerous advantageous qualities such as anti-cancer, anti-inflammatory, antioxidant, and antimicrobial properties. This allows nanoparticles to be formed with biomedical attributes [92]. Numerous research articles on the mycosynthesis of nanosilver have been published and continue to grow in number including El-Moslamy et al. [93], Ammar et al. [94], and Awad et al. [95], using endophytic *Trichoderma harzianum* SYA.F4, *Pichia kudriavzevii* HA-NY2 and *Saccharomyces* uvarumHA-NY3, and *Aspergillus niger* strains. Several recent reviews have highlighted the biogenic nanoparticle production of this element using fungal cultures [82,96,97,98].

During the production of silver nanoparticles (AgNPs) using fungus, the AgNPs are mostly created extracellularly, employing fungal extracts or culture supernatants, rather than inside the fungal cells or on the cell surface [99]. The procedure involves cultivating the fungal strain with appropriate growth conditions, preparing the fungal cell filtrate or culture supernatant, and adding it to an aqueous solution of silver nitrate (AgNO_3_) [100]. This commences the reduction of Ag^+^ ions, which results in the creation of AgNPs. The reaction mixture is then incubated at room temperature, allowing AgNPs to be synthesized extracellularly using the reducing and stabilizing biomolecules found in fungal extracts [101]. Monitoring the formation of AgNPs involves observing color changes in the reaction mixture and characterizing the nanoparticles using techniques like UV–vis spectroscopy, XRD, and TEM. The extracellular synthesis method is favored for its environmental friendliness and simplification of the process by eliminating extraction and purification steps [102].

#### 2.2.3. Green Synthesis of Silver Nanoparticles Using Algae

Algae play a significant role in producing silver nanoparticles using a fast and inexpensive procedure. The surface of algal cells carries the negative charge that speeds up the nucleation and growth of crystals, which accelerates the overall process [82]. The extraction technique for producing silver nanoparticles from algae begins with washing, drying, and grinding the algae biomass to increase surface area. Bioactive components such as polysaccharides and proteins are then extracted from algal biomass using solvent extraction or microwave-assisted extraction methods. These chemicals serve as reducing and stabilizing agents throughout the synthesis process. The extracted bioactive chemicals are then combined with a silver precursor solution, commonly silver nitrate, to begin the reduction of silver ions and the production of silver nanoparticles. Characterization methods such as TEM, SEM, XRD, and FTIR are used to examine the characteristics of the produced nanoparticles. This green synthesis process demonstrates the potential of algae in nanoparticle manufacturing, providing a sustainable and ecologically friendly approach with numerous applications in a variety of fields [103].

El-Rafie et al. [104] used water-soluble polysaccharides from four marine macro-algae (*Pterocladiacapillacae*, *Janiarubins*, *Ulva faciata*, and *Colpmeniasinusa*) to produce silver nanoparticles. These polysaccharides served as reducers and stabilizers. Öztürk et al. [105] used an extract of red algae *Gelidium corneum* as a reducing agent to produce silver NPs in a greener procedure. In addition, Fatima et al. [106] utilized red algae *Portieriahornemannii* for the efficient production of silver nanoparticles.

#### 2.2.4. Green Synthesis of Silver Nanoparticles Using Bacteria

Green synthesis mediated by microbes holds a distinct place despite the many natural resources for the green production of nanoparticles [107]. The simplicity of manipulation, high growth percentage, ease of culturing, and potential to grow in ambient temperature, pH, and pressure, are all reasons for “bacterial preference” for NP production [108,109]. Various groups of microorganisms can be categorized based on where they synthesize NPs. The extraction technique for producing silver nanoparticles using bacteria is a biogenic strategy that utilizes the advantage of bacterial cells’ specific capabilities to decrease silver ions and enhance nanoparticle synthesis. Initially, an effective bacterial strain is chosen based on its capacity to generate enzymes or proteins that can function as reducing agents. The chosen bacteria are cultivated in a nutrient-rich medium under regulated conditions to stimulate growth and metabolic activity. When the bacterial culture reaches a specified growth phase, it is exposed to a silver precursor solution, usually silver nitrate, which is subsequently reduced by the bacterial cells to produce silver nanoparticles. UV-vis spectroscopy is commonly used to monitor the reduction process and track nanoparticle production. After production, the silver nanoparticles are removed from the bacterial cells using centrifugation or filtering. TEM, SEM, XRD, and FTIR are used to characterize the size, shape, crystallinity, and chemical composition of the produced nanoparticles [110].

Bharti et al. [111] synthesized AgNPs using extracellular *Thiosphaera pantotropha*, while intracellular production of AgNPs utilizing *Rhodococcus* spp. was conducted by Otari et al. [112]. Table 1 shows some AgNPs synthesized using diverse bacterial strains.

#### 2.2.5. Synthesis of AgNPs Using Bioflocculants

Bioflocculants are natural, biodegradable compounds derived from natural sources such as microbes, plants, and algae [118]. They are essential as effective and eco-friendly methods of nanoparticle production. During synthesis, bioflocculants function as reducing and stabilizing agents for ions and nanoparticles by promoting aggregation and settling of particles to assist in the removal of pollutants from water sources [119]. Bioflocculants stabilize nanoparticles by using functional groups in their structure, such as hydroxyl, carboxyl, and sugar derivatives [120]. These groups interact with the nanoparticles’ surface to avoid agglomeration and ensure their stability. They have a substantial influence on both the shape and size of nanoparticles. Bioflocculants can alter the morphology of nanoparticles, resulting in the formation of particles with certain forms such as spherical or rod-like structures. In addition, bioflocculants can affect the size distribution of nanoparticles, impacting their characteristics and applications.

To produce bioflocculants, microorganisms such as bacteria, fungi, and actinomycetes are isolated and screened for their bioflocculant production capability [121]. Once identified, these microorganisms are grown in a medium containing carbon and nitrogen sources to promote their development and bioflocculant production. During fermentation, which may take several days, microorganisms produce the bioflocculant. After fermentation, the bioflocculant is recovered from the culture broth using centrifugation and precipitation with solvents such as ethanol. Additional purification techniques, such as solvent extraction, dialysis, or chromatography, are used to produce a pure bioflocculant product. Analytical methods are also utilized to characterize the bioflocculant, determining its content, structure, and characteristics. To improve bioflocculant yield and performance in a variety of applications, production factors such as microbe selection, growing conditions, and purification processes can be adjusted [118].

Several research have also reported the production of Ag using natural sources [122]. However, few studies have gone through the fabrication of silver NPs utilizing bioflocculants. Therefore, in this review, we will concentrate on certain of the latest work published on the production of silver NPs utilizing bioflocculants from the year “January 2015 to December 2023”. Adebayo-Tayo et al. [42] employed bioflocculant from *B*. *subtilis* to produce AgNPs. To achieve this, about 10 mL of the bioflocculant was added to 10 mL of 10 mM AgNO_3_ solution. The resultant mixture was stored at an ambient temperature in the dim area for 24 to 27 h. Controls were made and studied utilizing similar testing conditions but without the addition of silver nitrate. The change in color from lemon yellow to chocolate brown implies the production of AgNPs. However, under the same conditions, no change in color was detected in the sample control (bioflocculants without silver solution).

Adebayo et al. [123] researched the synthesis of AgNPs employing bioflocculant extracted from a combination of *Bacillus*, *Klebsiella*, *Acinetobacter*, and *Providencia* species. To synthesize AgNPs, a reaction vessel with 25 mL of 1 mM AgNO_3_ was made, and then a cell-free bioflocculant of 1 mL was added separately. The mixture was then exposed to sunlight and the color change was visually monitored to observe the formation of AgNPs.

Another study performed by Hassan et al. [124] described the production of silver NPs using bioflocculant derived from *Psychrobacter cibarius* H41A KF207755. The researchers noticed the solution’s color change from yellowish to brown which indicates the creation of the Ag nanoparticles.

Ajani and colleagues synthesized silver nanoparticles using the bioflocculant extracted from *Streptomyces* sp. HDW7 and *Nocardia* sp. OX5. The microbial-flocculant was utilized to produce AgNPs by employing its supernatant. To synthesize the nanoparticles, a solution of 2 mM AgNO_3_ was mixed with 1:1 *(v/v)* of each bioflocculant. The resulting solution was left in a dark place overnight. The control was AgNO_3_ without the addition of a bioflocculant. The transformation in color from pale AgNO_3_ to yellowish was observed by Ajani et al. [125].

The work by Muthulakshmi et al. [126] detailed the bioflocculant that was isolated from the fermented liquid of *Bacillus* sp. The liquid obtained from fermentation was subjected to centrifugation at a speed of 12,000 rpm for 30 min at ambient temperature. The resulting liquid above the sediment, known as the supernatant, was collected and utilized as a substance that reduces and binds during the production of silver NPs. Researchers observed the color change which was indicative of the successfully synthesized nanoparticles which were later confirmed using UV–vis spectroscopy.

Sajayan et al. [127] described the production of silver NPs using bioflocculant derived from *Bacillus cereus*. A 1 mM solution of AgNO_3_ was made in a 100 mL Erlenmeyer flask. In the same flask, equal amounts of the produced bioflocculant and silver NO_3_ solution (in a 1:1 ratio, *v*/*v*) were combined. The mixture was then placed in a dark environment and incubated at 28 °C for 96 h. During this time, the flask was monitored for a color transformation from yellow to brown which indicated the formation of silver nanoparticles. After the incubation period, the produced silver NPs were assembled by subjecting the mixture to centrifugation at a speed of 10,621× *g* and air-dried to produce a powder. The synthesis of NPs by bioflocculant extracted from *Bacillus cereus* was validated and confirmed by spectrophotometry of the solution in the UV–vis range.

## 3. Factors Influencing the Production of Silver NPs

The synthesis, characterization, and application of NPs are controlled by various factors. According to several researchers, the nature of the nanoparticles synthesized can vary based on the adsorbable type and the function of the catalysts throughout the production procedure [26,128]. Certain researchers have also observed that the synthesized NPs exhibit dynamic behavior, showing different symptoms and implications over time and in different environments [129]. Other significant parameters that influence nanoparticle synthesis include solution pH, temperature, the concentration of extracts and raw materials utilized, size, and the procedures used during the synthesis process [130]. Below are some significant factors that have a dominant impact on the production of silver NPs.

### 3.1. Effect of Starting Time and pH on AgNPs

pH is one of the important parameters that need to be optimized when assessing nanoparticle performance. Furthermore, it serves a vital function in preventing the formation of extra site product which acts like an impurity in the desired product [131]. Several studies have shown that alkaline pH levels lead to the formation of small nanoparticles, whereas acidic pH levels result in the production of larger NPs. Other researchers stated that the texture and size of the produced nanoparticle are caused by the medium solution’s pH [132]. Thus, by adjusting the pH of the broth solution, the nanomaterial size can also be adjusted.

The effect of time on nanoparticle (NP) formation is an important factor that influences the properties and behavior of the produced nanoparticles. Time is critical to the production and development of silver nanoparticles (AgNPs). Longer reaction durations result in the progressive creation of AgNPs, as demonstrated by color changes in the solution, increased particle size, and silver nanocrystal aggregation [133]. Furthermore, the size of AgNPs was shown to increase with reaction time, indicating a clear link between time and nanoparticle size [133]. Furthermore, the crystallite size of NPs tends to decrease with longer reaction periods, indicating the possibility of lower crystallite sizes with longer reaction durations [134]. AgNPs’ stability and toxicity are also affected by time, with storage conditions influencing aging and physicochemical qualities, with possibly harmful consequences. Monitoring the development of nanoparticles over time is critical for assuring their integrity, improving their characteristics, and determining their safety in diverse applications. Understanding and managing the temporal elements of NP synthesis is critical for realizing nanoparticles’ full potential in a variety of applications [135].

Fu et al. [136] deliberate the effect of stirring time and pH value on the production of silver nanoparticles. The experiment was conducted in two stages. During the initial phase, AgNO_3_ and Na_3_C_6_H_5_O_7_ were mixed for varying durations of 0 to 60 min. In the second stage, sodium borohydride was added to initiate the production of AgNPs. The results indicated that the stirring time during the first stage influenced the characteristics of the AgNPs produced. After analyzing all of the synthesized AgNPs after 120 min, it was determined that an agitation period of 10 min in the initial stage provided the optimum conditions for subsequent AgNP production. AgNO_3_ and trisodium citrate were successfully added and disseminated within the mixture when the mixing duration was less than 10 min. If the agitation time was reduced to 10 min, the reaction reagents were at risk of being lost. As a consequence, the ideal reaction time was 10 min for shaking in the initial stage, followed by 120 min for AgNP formation in the second stage.

The researchers also investigated how pH influences AgNP behavior. They used citric acid and sodium hydroxide to alter the pH of the AgNP solution from 4 to 12. Absorption spectra were obtained in the range of 300–600 nm wavelength. The Ag nanoparticles pH of the colloidal medium was initially set at 8. The reported absorbance values dropped when the mixture was exposed to an acidic condition. In an alkaline environment, however, the absorbance values of AgNPs increased. This behavior can be attributed to AgNPs’ inability to survive in acidic or neutral conditions, preferring an alkaline condition. Earlier research has shown that the level of pH has a substantial impact on silver nanoparticles by modifying their charge surface and hence incidentally impacting their aggregation and stability [137].

Silver nanoparticles are particularly unbalanced in neutral and acidic conditions, according to the literature. AgNPs gain more prominent surface charges under these conditions, resulting in greater aggregation and larger particle size. The shifting of peak absorption in the spectroscopy of surface plasmon resonance may account for the lower absorbance values of silver NPs. The work by Iqtedar et al. [138] supported the optimum production of silver NPs at pH 8.0. Another work by Elsayed et al. [139] observed that an optimum pH of 7.0 is effective in the formation of silver nanoparticles.

### 3.2. Effect of Temperature

In any production of NPs, the temperature is widely recognized as an important factor. Temperature is a crucial factor in the size, shape, and yield of nanoparticles [28]. The physical technique necessitates a higher temperature, above 350 °C, while the chemical method requires temperatures below 350 °C [140]. Nanoparticle fabrication using green procedures usually need a temperature of less than 100 °C [141]. The temperature of the reaction medium defines the character of the nanoparticles [142]. The increase in reaction temperature results in a reduction in nanoparticle size, whereas a decrease in temperature results in an increase in nanoparticle size [143].

Saeed et al. [144] examined the impact of temperature on the synthesized silver NPs at three different temperature levels, ranging from 28 to 45 °C. The findings revealed that the culture supernatant exhibited the highest UV–visible absorption at 37 °C, which remained consistent. This suggests that at 37 °C, there was a greater amount of kinetic energy present in the silver nanoparticles, resulting in a more stable and faster production of AgNPs. The particle size of the produced AgNPs has a peak due to the effect of temperature because of the intricate relationship between temperature and the growth of silver nanoparticles [145].

Another report by Liu et al. [146] examines the impact of dissimilar temperatures. It was found that at temperatures starting from 40, 60, and 90 °C, the particle sizes were 17.6 ± 4.5 nm, 15.3 ± 3.7 nm, and 7.8 ± 2.3 nm, respectively.

In a study by Vivekanandhan et al. [147], the performance of silver NPs at various temperatures was investigated by exposing liquids containing *C*. *tropicum* and *F*. *oxysporum* to AgNO_3_ within the temperature range of 25 °C to 30 °C. The rate at which silver nanoparticles formed was found to be influenced by the temperature of incubation, with higher temperatures resulting in faster particle growth. The impact of temperature was estimated using a micro-scan reader, and it was observed that a rise in temperature from 25 °C to 30 °C led to a rise in the production and absorption of silver nanoparticles by *C*. *tropicum* and *F*. *oxysporum*. At the lower temperature of 25 °C, the majority of silver nanoparticles were smaller in size. Upon further incubation at 30 °C and increased concentration, the quantity of smaller particles decreased, giving rise to the formation of larger particles exhibiting distinct shapes typical of silver nanoparticles. This observation establishes a direct correlation between the solutions’ absorbance and temperature, as evidenced by the equation derived from the experimental data.

### 3.3. Effect of Pressure on Nanoparticle Formation

Pressure has also been reported as a crucial factor in nanoparticle production [148]. The shape and magnitude of the produced nanomaterial are impacted by the tension that is applied to the media reaction [140]. In another work by Zikmund et al. [149], reports state that during the silver NP fabrication, the pressure was observed to increase when the particle size increased. The study discovered clear relationships between the aggregation distance chamber and mean particle size. Another study on the structural behavior of AgNPs discovered a size of 10 nm, indicating that as pressure increases, the size of the nanoparticles also increases [150]. Another work on AgNPs for fluorescent nanocomposites using magnetron high-pressure sputtering discovered that the size of the nanoparticles decreases with pressure [149], which contradicts previous studies where the nanoparticles reportedly increase with pressure [151]. According to the reports, the relationship between pressure and nanoparticle size is complex and depends on multiple influences such as the type of nanoparticle, the synthesis method, and the pressure range. Due to this, a more extensive study is required to properly comprehend the relationship between pressure and nanoparticle size.

### 3.4. Particle Size and Shape

Particle size is crucial when it comes to assessing the NPs’ properties. For example, when nanoparticles reach the nanometer scale, their melting point decreases [91]. The energy of nanoparticles of various configurations is similar, which makes the process of shape transformation simple [20]. The type of energy frequently employed during nanomaterials investigation results in a change in the nanoparticle’s structure [20]. The dynamic nature and form of formed NPs have a substantial influence on their chemical characteristics [152].

Ahmad et al. [153] revealed a rounded-form silver NP with a normal particle size of 15–25 nm. Additionally, AgNPs were found to be uniformly dispersed without any evidence of clustering or aggregation. Dynamic light scattering (DLS) measurements were used to determine particle size and distribution. The DLS observations revealed that the biogenic silver NPs were from 10 to 20 nm, with the majority of particles clustered around 15 nm. Some of the optimized parameter during synthesized nanoparticles is given in Table 2 below.

## 4. Characterization of the Synthesized AgNPs

Understanding the nanoparticles’ physical and chemical characteristics is crucial for several reasons, including the way they behave, circulation in biological systems, safety, and effectiveness [159]. Consequently, it is necessary to analyze the AgNPs to investigate the functionality aspects of the produced particles. Many analytical procedures, such as UV–visible spectroscopy, X-ray diffractometry (XRD), Fourier transform infrared spectroscopy (FT-IR), X-ray photoelectron spectroscopy (XPS), dynamic light scattering (DLS), scanning electron microscopy (SEM), transmission electron microscopy (TEM), and atomic force microscopy (AFM), are employed for characterization [160]. Numerous authoritative publications have explained the concepts and possible uses of the aforementioned methods for characterizing AgNPs. However, the following section provides a simplified overview of the fundamental procedures employed for AgNP characterization.

### 4.1. UV–Visible Spectroscopy

UV-vis spectroscopy is a valuable and dependable method for analyzing synthesized nanoparticles, particularly in the case of AgNPs [161]. It allows for the monitoring of AgNPs’ synthesis and stability by exploiting their unique optical properties and interaction with specific light wavelengths. UV-vis spectroscopy offers numerous advantages, such as its speed, simplicity, sensitivity, selectivity for different types of nanoparticles, and the fact that it needs just a short measurement time without the need for standardization when characterizing colloidal suspensions of particles [162]. In AgNPs, the conduction and valence bands are located at nearly the same energy, which allows for the free circulation of electrons [163]. This results in surface plasmon resonance (SPR), where the joint oscillation of molecules in the Ag nanoparticles resonates with the photon wave, leading to band absorption. AgNP absorption is impacted by factors such as the particle size, the surrounding chemical environment, and the dielectric medium [145]. The presence of a peak related to SPR, which represents this collective electron oscillation, has been well-documented for many metal NPs starting from 2 to 100 nm in size. UV–visible spectroscopy has been successfully used to observe the synthesized silver NP stability through biological procedures for over 12 months. In various studies, the presence of a plasmon resonance surface peak at an identical frequency was consistently observed employing ultraviolet-visible spectroscopy [164].

### 4.2. X-ray Diffraction

X-ray diffraction (XRD) is an essential procedure that can identify crystalline structures at the atomic level [165]. It is a non-destructive practice that shows promise for characterizing both organic as well as inorganic crystalline substances. This technique has diverse applications across various fields such as geology, polymers, environment, pharmaceuticals, and forensics, allowing phase identification, quantitative analysis, and the determination of structural imperfections [164]. Additionally, XRD has expanded its applications to the characterization of nanomaterials and their properties [166]. The idea behind X-ray diffraction is Braggs’ law, where X-rays are elastically scattered at wide angles [167,168]. X-ray diffraction (XRD) is also employed for the qualitative identification of different compounds, quantitative resolution of chemical species, measuring crystallinity [169], studying isomorphous substitutions [170], determining particle sizes [171], and more. When X-rays interact with a crystal, they produce multiple diffraction patterns that reveal the chemical and physical features of the crystal shape [172]. In the case of a powdered sample, the diffracted beams are scattered by the sample and reveal structural and physicochemical properties. As a result, XRD can analyze the structural features of various materials, including inorganic catalysts, superconductors, biomolecules, glasses, polymers, and others [173]. The analysis of these materials heavily relies on the interpretation of diffraction patterns. The identification of each material can be accomplished by comparing the diffracted beam with its distinctive diffraction pattern to the standard records available in the Committee Power Joint Standards Diffraction (CPJSD) database. Moreover, pattern diffraction can signify the occurrence of impurities in the sample, allowing XRD to be employed in the characterization and identification of large and tiny materials, forensic specimens, manufacturing samples, and chemical substances [174].

### 4.3. Dynamic Light Scattering (DLS)

The study of biological activities utilizing scattering radiation procedures relies on the physicochemical characterization of prepared nanomaterials, which is an important factor [164]. DSL is a technique that uses light interaction with particles to explore the distribution size of tiny materials in suspension that ranges from small to one nanometer [175]. DLS is a commonly employed technique for characterizing nanoparticles [176]. It has the potential to determine the scattered light from suspended nanoparticles as a beam of light moves through a matrix, primarily relying on Rayleigh scattering [176]. The hydrodynamic size of NPs can be calculated by examining the change of scattered light intensity over time [177]. Characterizing nanomaterials in solution is crucial for evaluating their toxic potential [164], and DLS is most commonly employed to evaluate particle size and particle size distribution in solution form [79]. It is worth mentioning that the size achieved via DLS is often larger than that produced through TEM, possibly due to the impact of the principle’s Brownian motion. DL scattering is a non-destructive technology that delivers the mean size of nanoparticles scattered in solution and has the benefit of investigating a bigger number of particles. Nevertheless, it also has some limitations specific to the sample being analyzed [178].

### 4.4. Fourier Transform Infrared (FT-IR) Spectroscopy Analysis

FT-IR spectrometry is defined as a highly accurate and reproducible method that can produce a favorable signal-to-noise ratio [179]. It is capable of detecting small absorbance changes as low as 10^−3^, making it ideal for difference spectroscopy, which allows the differentiation of active residues from the absorption background of a protein as a whole [164]. FT-IR is useful in determining whether biomolecules participate in the production of nanoparticles, particularly in academic and industrial research [178]. This technique was additionally utilized for the investigation of nano-scale substances, including the affirmation of functional compounds directly attached to Ag, carbon nanotubes, graphene, and AuNPs, as well as interactions that occur between the substrate and the enzyme during a catalytic process [180]. Another advantage of the FT-IR technique is that it is a non-invasive technique [181]. Moreover, FT-IR spectrometers offer several benefits over dispersive ones, such as fast data collecting, producing a powerful signal, low ratio of noise to signal, and minimal sample heat-up [164]. Attenuated total reflection (ATR)-FTIR spectroscopy is a recent advancement in FT-IR methodology. ATR-FT-IR is capable of determining the chemical features of the surface polymer, and the preparation of the sample is comparatively straightforward compared with traditional FT-IR [182]. Consequently, FT-IR is an appropriate, beneficial, non-invasive, affordable, and easy approach to identifying the biological functional compounds involved in reducing silver NO_3_ to Ag [183].

### 4.5. Scanning Electron Microscopy

Nanosciences, such as nanotechnology, have served as pivotal forces in the development of numerous high-resolution microscopy techniques that employ a strong probe to electrons on a small scale [184]. One such technique is SEM, which is a surface imaging method that can effectively examine the size of particles, distribution size, nanomaterial structure, and morphology surface of produced materials at the small and nanoscales [185]. By using SEM, we can examine particle morphology and create a histogram from images, either by manually counting and measuring the particles or by utilizing specialized software. When paired with energy-dispersive X-ray spectroscopy (EDX), scanning electron microscopy (SEM) can be used to examine the morphology and chemical composition of silver materials [185]. However, SEM is unable to resolve internal structures, even though it can still offer helpful data on purity particles and the amount of agglomeration [186]. Modern high-resolution SEMs are capable of identifying nanoparticle morphology at sizes of less than 10 nm [178].

### 4.6. SEM-EDX Analyser

SEM-EDX is frequently used in conjunction with SEM to perform elemental analysis. EDX detects the characteristic X-rays emitted by the sample and provides explanations based on the elemental composition of the nanoparticles. By measuring the energy and intensity of the X-rays, EDX can identify the elements present and estimate their relative abundances [187].

### 4.7. Transmission Electron Microscopy

Transmission electron microscopy (TEM) is an essential and frequently utilized method for analyzing nanomaterials [188]. It plays a crucial role in obtaining quantitative data regarding grain size, distribution size, and structure [189]. The level of magnification achieved in TEM primarily depends on the gap between the objective lens and the specimen, as well as the distance between the objective lens and its plane image [189]. Compared to scanning electron microscopy (SEM), TEM offers two advantages: superior spatial resolution and the ability to perform more analytical measurements [190]. However, there are some limitations to TEM, including the necessity for an intense vacuum and a tiny sample portion [191]. One vital aspect of TEM is the time-consuming nature of sample preparation, which is crucial for acquiring high-quality images [192]. Therefore, utmost attention must be given to sample preparation to obtain the best possible images.

### 4.8. Thermogravimetric Analysis

TGA is a characterization technique employed to investigate the thermal properties of materials [187]. It entails subjecting a sample to measured temperature changes while determining its weight as a function of temperature or time. TGA gives useful information regarding a material’s thermal stability, decomposition behavior, moisture content, and composition [193]. During TGA analysis, the sample is typically heated in a controlled environment, such as a furnace, while its weight is continuously monitored [194]. As the temperature increases, various thermal events can occur, leading to weight changes in the sample. These events include dehydration, decomposition, oxidation, phase transitions, and volatilization. It can be used to determine important parameters such as the onset temperature of thermal events, the rate of weight change, the presence of multiple decomposition steps, and the residual mass of the sample after complete decomposition [187]. TGA is useful in various departments, including materials science, chemistry, polymers, pharmaceuticals, and environmental analysis [195]. It provides crucial information for understanding the thermal behavior and stability of materials, aiding in material selection, process optimization, quality control, and product development [195].

## 5. Physicochemical Properties of the Produced AgNPs

Adebayo and colleagues reported UV–vis spectroscopy at 570 nm and 460 nm for AgNPs synthesized from a consortium of *Bacillus*, *Klebsiella*, *Acinetobacter*, and *Providenci*. For the consortium, FT-IR peaks of AgNPs were observed at 3525, 3284, and 1179 cm^−1^, corresponding to alcoholic groups, amide groups, and alcohol, respectively, and 3611, 3307, and 2492 cm^−1^, corresponding to aromatic primary amines, carboxylic acids (CA) and amines, respectively [123].

Manivasagan et al. [196] revealed that the XRD study confirmed the natural crystallinity of the silver nanoparticles, with the peaks at 2*θ* values of 38.25°, 44.37°, 64.60°, and 77.64°, corresponding to 111, 200, 220, and 31 planes of the face-centered cubic (fcc) structure of silver, respectively. FT-IR study discovered the occurrence of functional groups on the surface of the AgNPs, which may contribute to their stability and reactivity. UV–visible spectroscopy showed a characteristic absorption peak at around 418 nm, indicating the presence of AgNPs in the solution. UV–vis absorption spectra displayed the broad surface plasmon resonance at 420 nm. The analysis of the FT-IR spectrum indicates that there are peaks of absorption at 3428, 2071, 1634, 1067, and 687 cm^−1^. These peaks were observed in the biosynthesis of silver nanoparticles and are attributed to O–H stretching (3428 cm^−1^), C–O (2071 cm^−1^), N–H bend (1634 cm^−1^), C–N (1067 cm^−1^), and C–Br (687 cm^−1^). To prevent the NPs from coagulating due to Van der Waals forces, nanoparticles need to be stabilized during the formation process. The data from the FT-IR analysis supports the occurrence of O–H stretching (3428 cm^−1^) which could be liable for the reduction of ions metal into their corresponding NPs.

Nabil-Adam and Shreadah [197] reported the results for EDX which revealed the occurrence of carbon, oxygen, chlorine, sulfur, and sodium in addition to Ag. Yumei et al. [198] reported the X-ray diffraction (XRD) pattern of the AgNPs to display four prominent peaks at 38.2, 44.3, 64.4, and 77.5 in the 2-theta range. These peaks correspond to the (111), (200), (220), and (311) crystallographic planes of silver. The presence of silver in the nanoparticles was further confirmed by surface plasmon resonance (SPR) bands with the aid of a UV–vis spectrometer. The observation indicated that the AgNPs had a robust SPR transition and absorbed radiation within the 400–500 nm visible range. The FT-IR spectral analysis indicated the existence of the primary absorption peaks at 3098, 1604, and 1078 cm^−1^, which were caused by O–H stretching, C–H stretching, and C=O stretching. The change in the position of the C=O and –OH absorption peaks before and after AgNP production validated the role of the bioflocculant in reducing Ag^+^ ions and stabilizing AgNPs as capping agents. Additionally, the occurrence of Ag–O van der Waal interactions between 472 and 594 cm^−1^ was observed, which was not apparent in the bioflocculant extracted from the *Arthrobacter* sp. spectrum.

In the study by Nabil-Adam and Shreadah [197], the AgNP size was determined to be 19.66 to 26.96 nm. Manivasagan et al. [196] synthesized AgNPs using a bioflocculant produced by a bacterial *Streptomyces* sp. MBRC-91. The TEM images showed that the synthesized AgNPs were spherical with an average size of 35 nm. In the study by Yumei et al. [198] silver nanoparticles using a bioflocculant extracted from *Arthrobacter* sp., the geology of the silver nanoparticles (AgNPs) was examined through the capture of high-resolution transmission electron microscopy (HRTEM) images. The particle size distribution was then determined using Image J software to analyze the (HRTEM) images. The obtained results indicated that the nanoparticles possessed a generally spherical shape, with diameters of 9 to 72 nm.

## 6. Application of Silver Nanoparticles

**Figure 3:**
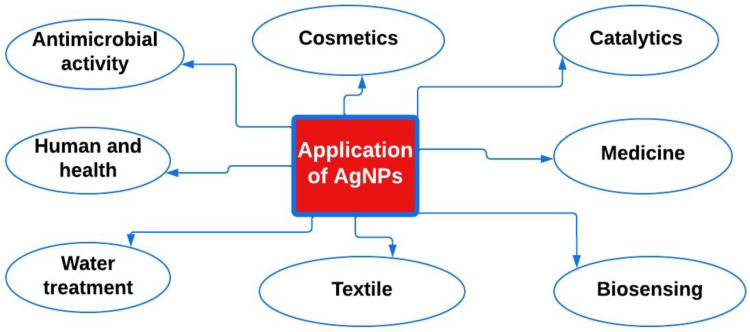
Applications of silver nanoparticles [23]. Created with Lucidchart.

Silver NPs are recognized for their unique features and are frequently employed in several settings, involving water treatment, healthcare, and preservation of food, as well as nature [199]. Many reviews and book chapters have explored the different uses of AgNPs [200]. This article focuses specifically on their applications in wastewater treatment and biomedical fields, including their antimicrobial activity, and antibacterial and anticancer properties. The article reviews earlier foundational works before offering the newest information. A representative illustration of the numerous silver NP applications is shown in Figure 3.

### 6.1. Antimicrobial Activity of Silver NPs

Various accredited bodies, such as USFDA, USEPA, Korea’s testing body, and SIAA of Japan Institute of Research, have approved products that are made using silver nanoparticles [201]. These products, which incorporate silver sulfadiazine, are known for their antimicrobial and antibacterial potential and are used in burn treatments to prevent infections. Silver nanoparticles have become more prevalent within nanotechnology applications and are now found in various products produced for public consumption, including acne creams and deodorizing sprays [202]. The effectiveness of the antimicrobial properties of silver nanoparticles is affected by elements like size, pH levels, and tonic strength, as well as the capping agent used. Current work has publicized that combining silver NPs with some antibiotics like ampicillin, amoxicillin, and chloramphenicol can result in improved antimicrobial activity [203]. However, some reports suggest that combining silver nanoparticles with amoxicillin or oxacillin antibiotics can lead to an antagonistic interaction [204], while others have suggested that the combination may improve therapeutic activity [23]. Ghiuță et al. [205] observed the antimicrobial effectiveness against *C*. *albicans* to be significantly greater in comparison to the control antibiotic. The Fluconazole disks alone showed minimal to no impact on the *C*. *albicans* strain, but when used together with AgNPs, a measurable zone of inhibition was observed. The positive impact of AgNPs on the antibiotics’ effectiveness was determined by calculating the percentage increase in antibacterial effect.

Natarajan [206] used well and disk diffusion methods to examine the effectiveness of AgNPs in combating different microorganisms that contribute to environmental degradation. The researchers measured the diameter of the zone surrounding each well and disk where the growth of microorganisms was inhibited. The results indicated that AgNPs exhibited the greatest antimicrobial activity against *Bacillus subtilis*, with *Pseudomonas aeruginosa* and *Acidithiobacillus ferredoxin dans* following closely behind. The bactericidal properties of AgNPs were found to be consistently effective across the various bacterial strains tested.

Other work by Saeed et al. [144] reported AgNPs with a high surface area-to-volume ratio to be particularly effective at inhibiting the growth of bacteria. The researchers used bacterial-mediated silver nanoparticles in an agar well diffusion experiment to assess their antibacterial properties. They examined the antimicrobial activity against 13 different harmful bacteria and quantified the clear zones surrounding the wells. Their findings indicated that silver nanoparticles helped treat multidrug-resistant bacteria and other harmful microorganisms.

### 6.2. Antibacterial Activity of AgNPs

AgNPs are potential alternatives to antibiotics for fighting bacterial infections, as they can overcome bacterial resistance [207]. As a result, the development of AgNPs as antibacterial agents is critical. AgNPs stand out among promising nanoparticles because of their substantial surface-to-volume ratios and surface crystalline structure, which makes them effective against bacteria [208]. A significant study conducted by Sondi and Salopek-Sondi confirmed the antimicrobial properties of silver NPs against *Escherichia coli*. The study found that *E*. *coli* cells treated with AgNPs showed the accumulation of AgNPs in the cell wall, resulting in the formation of “pits” and eventual cell death [209] Gudikandula and Charya Maringanti [210] have shown that silver nanoparticles produced using biological means using fungi (*Pycnoporus* sp.) exhibit stronger antibacterial properties in comparison to silver nanoparticles produced through chemical methods. The experiments included testing the antibacterial activity against both gram-positive and gram-negative bacteria.

Verma and Maheshwari [211] found that the nanocrystalline chlorhexidine silver (CHX Ag) compound had significant antibacterial action on Gram-positive/negative and methicillin-resistant *Staphylococcus aureus* (MRSA) strains. The MICs of nanocrystalline Ag (III) CHX were significantly lower than those of the ligand (CHX), AgNO_3_, and silver sulfadiazine.

Tang and Zheng [212] reported that smaller AgNPs with higher surface-to-volume ratios exhibited greater antibacterial activity compared to larger particles. For example, they observed that smaller AgNPs, which have strong antibacterial properties, outperformed larger AgNPs [213]. Their findings demonstrated that AgNPs with a size of 9 nm had approximately nine times higher antibacterial activity compared to their larger counterparts measuring 60 nm. The researchers speculated that the primary explanation for the observed antibacterial effects of silver nanoparticles is the oxidation (Ag^+^) of the smaller AgNPs, as they are highly susceptible to oxidation. To test this hypothesis, zerovalent (reduced) AgNPs were synthesized in an oxygen-free environment and compared their antibacterial activity to AgNPs that had been partially oxidized. Researchers observed that the reduced AgNPs had minimal toxicity against *E*. *coli* cells, whereas the oxidized AgNPs demonstrated significant inhibition of cell survival and depletion of APT content. Saravanan et al. [214] reported silver nanoparticles (AgNPs) to exhibit antibacterial effects against multidrug-resistant (MDR) strains like *Salmonella typhi* and *Staphylococcus aureus*.

### 6.3. Antifungal Activity of AgNPs

The usage of antifungals for silver nanoparticles (AgNPs) has been thoroughly researched and confirmed in several research publications. AgNPs have demonstrated substantial antifungal activity against Candida species, particularly *Candida albicans*, making them a prospective treatment for fighting fungal infections [215]. Studies have compared the antifungal effectiveness of AgNPs to traditional antifungal medicines such as Amphotericin B, Fluconazole, Grisofulvin, and Itraconazole, revealing that AgNPs are effective antifungal agents. AgNPs effectively suppress *Candida albicans* growth, with MIC values ranging from 0.125 to 64 μg/mL. Furthermore, AgNPs were discovered to be more powerful than standard antifungal drugs, emphasizing their promise as a novel and effective antifungal therapy alternative.

Khatoon et al. [216] found that silver nanoparticles produced from tulsi leaf extract had considerable antifungal action, limiting the development and pathogenicity of Candida, an opportunistic human fungal infection. According to the researchers, the inhibitory impact may be caused by the degradation of fungal cell membrane integrity and virulence components.

### 6.4. Anticancer Activity of AgNPs

During our lifetime, approximately one out of every three individuals faces the risk of developing cancer [217]. Although numerous chemotherapy drugs are presently utilized for treating different types of cancer, their significant side effects, coupled with the laborious process of administering them through intravenous infusion, pose considerable challenges [164]. Hence, it is crucial to explore technologies that can minimize systemic side effects. Consequently, numerous scientists are involved in initiating the NPs as a substitute strategy to formulate treatments that specifically mark cell tumors [119,218].

Various research laboratories have employed different cell lines to investigate the potential of discovering novel molecules for combating cancer [218]. In this summary, we have compiled the findings from several laboratories which highlight the anti-cancer activity of these molecules by both *in vitro* and *in vivo* models. Gopinath and colleagues conducted a study to investigate the silver nanoparticles mechanism, and their findings revealed that the concentration of AgNPs influenced automatic cell death. They also found that there was a synergistic effect on apoptosis when using cells expressing uracil phosphoribosyltransferase (UPRT) and non-UPRT-expressing cells in the presence of fluorouracil (5-FU). The researchers observed that AgNPs not only induced apoptosis but also sensitized cancer cells in these experimental conditions [219].

Ahmad et al. [153] reported the biocompatibility of the silver nanoparticles to be used and evaluated using the J-774 cell line. The results of the cytotoxicity test, which varied depending on the dosage, indicated that both types of silver nanoparticles demonstrated excellent compatibility with cells within the dose range of 5–40 μg/mL after a 24 h incubation period. When exposed to concentrations above 50 μg/mL, the nanoparticles exhibited moderate cytotoxicity, resulting in a 75% survival rate for the cells. However, this concentration was still higher than the minimum inhibitory concentration required to inhibit the growth of the tested bacterial strains. Consequently, these findings suggest that the silver nanoparticles synthesized through this method can potentially serve as safe and effective antimicrobial agents.

### 6.5. Wastewater Treatment

Silver nanoparticles are widely studied and used in various applications, including water and wastewater treatment. Presently there is an increased interest in the use of AgNPs as an upcoming approach to conventional water treatment methods as a result of inimitable properties including great surface area, reactivity, and antimicrobial agents [220]. The ability of silver NPs to remove pollutants is one of the most interesting uses of silver NPs in water and wastewater purification [221]. Silver nanoparticles are effective in removing toxic metals such as lead, cadmium, and mercury from polluted water sources [222]. This is due to the high reactivity of AgNPs with metal ions, resulting in the formation of metal nanoparticles which can be easily removed through sedimentation or filtration [222]. In addition, silver NPs were found to be successful in eliminating organic contaminants such as dyes, pesticides, and pharmaceuticals from wastewater. The large surface area of AgNPs permits the increased adsorption of these pollutants, while their antimicrobial activity can also help to degrade them [223]. AgNPs can potentially be utilized as a disinfecting agent in water purification. The antimicrobial properties of AgNPs can help to inactivate bacteria, viruses, and other pathogens, making them a potential alternative to traditional disinfectants such as chlorine [224].

Adebayo-Tayo et al. [42] observed a decrease in the levels of chemical oxygen demand (COD), biological oxygen demand (BOD), total soluble solid (TSS), total dissolved solids (TDS), and turbidity after AgNPs synthesized from *Bacillus subtilis* B2 bioflocculant were applied. The results indicated the largest decreases in BOD, COD, TSS, and turbidity, with reductions of 33.33%, 71.44%, 83.70%, and 85.69%, respectively. Furthermore, AgNPs were more effective in removing metals in comparison with potassium aluminium sulfate (Alum). AgNPs also displayed antibacterial activity against the studied *E*. *coli* strains, resulting in a 100% reduction in the coliform count of the treated dairy effluent samples.

Gadkari et al. [225] stated the possible practice of AgNPs covered by the foam of polyurethane as an antibacterial water purifier. The researcher utilized a 1.5 L solution of the nanoparticles for a coating saturated with 20–25 cm foamy and an 8 mm width. Overnight, the foamy was submerged in a silver mixture. This covered foam was then tested for antibacterial filtering action. The bacteria concentration in the input water was approximately 1105–1106 colony-forming units (CFU)/mL, while no microbes had been discovered in the water outflow. Furthermore, no growth of bacteria was found in the foam beneath.

Liu et al. [226] discovered that silver nanoparticles were adsorbed onto a mesoporous silica matrix and used to eliminate ciprofloxacin in wastewater. The results showed that the AgNPs-silica composite had a high adsorption capacity and could remove up to 98% of ciprofloxacin from wastewater. Sharma et al. [157] reported that silver nanoparticles embedded in a chitosan/poly (vinyl alcohol) (CS/PVA) matrix were able to eliminate heavy metal ions from wastewater. The results showed that the AgNPs-CS/PVA composite had a high adsorption capacity for heavy metal ions, such as Cu (II), Pb (II), and Cd (II).

A study conducted by Mohammed [227] revealed that silver nanoparticles were used as a disinfectant for wastewater treatment. The AgNPs were added to the wastewater, and the disinfection efficiency was evaluated using *Escherichia coli* as the indicator organism. The results showed that the AgNPs effectively killed the bacterium and could be used as a disinfectant for wastewater treatment.

Another study by Bhatt et al. [221] used AgNPs to remove heavy metals and organic pollutants from wastewater. The results showed that AgNPs were effective in removing copper, lead, and zinc ions from wastewater, as well as reducing the levels of organic pollutants.

Allam et al. [228] examined the success of AgNPs in eliminating heavy metals from wastewater. The results showed that AgNPs were able to remove up to 90% of the heavy metals in the wastewater. Adebayo et al. [123] reported the successful elimination of all the microbes in fecally contaminated wastewater using AgNPs synthesized consortium bioflocculant, which had a concentration of 146 CFU/mL when the AgNPs were present at a concentration of 20 μg and diluted by a factor of 10^−5^. These findings indicate that AgNPs produced by a bioflocculant and free of cells, possess antibacterial properties and can be employed as an effective agent for purifying water.

The costs associated with employing silver nanoparticles (AgNPs) for wastewater treatment are numerous. While the material costs of AgNPs can be somewhat costly, advances in green synthesis techniques based on plants or microbes provide a more cost-effective option for production [229]. Despite the initial investment, AgNPs are viewed as a competitive alternative to existing wastewater treatment technologies because of their simplicity, adaptability, eco-friendliness, and overall cost-effectiveness. The capacity to regenerate and reuse AgNP-based materials after adsorbing pollutants improves their economic feasibility by increasing utility while decreasing treatment costs. Continued study into optimizing synthesis procedures, recovery methods, and regeneration techniques will be critical in increasing the cost-effectiveness of using AgNPs for wastewater treatment [145].

### 6.6. Dye Removal

Dyes are chemical substances that are used to add color to a variety of products, including textiles, paper, plastics, and inks. When dyes enter water bodies such as rivers, lakes, or groundwater, they can negatively affect human health along the environment [230]. Silver NPs have been shown to have the potential the eliminate dyes from effluent [231]. Multiple research projects have recently been initiated to assess the efficacy of Ag nanoparticles in eliminating various types of dyes from water [232,233]. A work by Marimuthu et al. [232] investigated the use of AgNPs in removing methylene blue dye from water. The study found that the nanoparticles were able to effectively remove the dye, with a removal efficiency of up to 95%. The researchers also found that the removal efficiency improved with rising concentrations of silver NPs. Another study by Ali et al. [234] investigated the use of silver nanoparticles in removing Congo red dye from water. The researcher testified that nanoparticles were successfully eliminated from the dye, with a removal efficiency of up to 98%. The researchers also found that the removal efficiency increased with increasing concentrations of silver nanoparticles and with increasing contact time between the nanoparticles and the dye.

Anil et al. [235] developed a 50 mg/L employed-solution standard for the dyes Congo and Methyl orange. About 50 mL of this solution was transferred to each of the four 100 mL conical flasks. Following that, each flask containing the dye solution received 1 mg of biosynthesized silver nanoparticles. The mixture was then ultrasonically mixed at a frequency of 20 kHz for 15 min. After mixing, a solution of 2 mL was removed at regular intervals. The samples’ optical densities (OD) were calculated at 415 nm for Methyl orange and 495 nm for Congo red. These findings were taken at hourly intervals starting from 0 h up to 12 h. After 24 h, a final OD measurement was taken. For analyzing the solution, a UV–vis spectrophotometer was utilized specifically at a wavelength of 495 nm. The percent degradation at every single time interval was performed. The researchers reported that the highest degradation of Congo red, with a degradation percentage of 87.36 ± 0.637% was achieved while degradation of Methylene orange was observed to be 71.20 ± 0.179%.

## 7. Cytotoxicity of Silver Nanoparticles

In general, it is critical to assess the detrimental impact of silver nanoparticles on cells to ensure the well-being of individuals, preserve the environment, adhere to regulatory standards, and make progress in our understanding of nano-toxicology [236]. In biomedical applications involving nanoparticles, biologically generated silver NPs were discovered to show toxicity influence towards many cell types through different mechanisms. These mechanisms include disruption of the mitochondrial membrane, alteration or induction of apoptosis (cell death), and the induction of DNA damage [237]. The use of AgNPs for their antiproliferative effect has potential applications in cancer chemotherapy, as they can affect cancerous cells in different ways [238]. This variation in reaction could be due to AgNPs interfering with cellular processes such as the induction of changes in chemistry cellular, and protein function in the cells, such as unfolded and agglomeration [238]. AgNPs are harmful to both mice of fibroblast NIH3T3 embryonic cells and MDA-MB-231 breast human cancer cells, inducing genotoxicity and cytotoxicity, as well as changes in cell shape and oxidative stress [239]. The harmfulness effect of silver NPs on cells is proportional to the silver nanoparticles concentration utilized. Multiple concentrations of AgNPs were investigated, and it proved that silver NPs did not affect cell viability at 50 μg/mL. However, at a concentration of 1000 μg/mL, the viability of the cell lines was significantly reduced [240].

Eghbalifam et al. [241] also revealed that the level of toxicity exhibited by AgNPs relies on their concentration. Nonetheless, even at very low concentrations, AgNPs possess antibacterial properties against prokaryotic organisms. The researchers observed also that AgNPs do not harm eukaryotic cells to the same extent as prokaryotic cells; likely due to the larger size and greater structural and functional redundancy of eukaryotic cells. As a result, bacterial cells are effectively targeted while no discernible detrimental effects are observed in eukaryotic cells.

## 8. Conclusions and Future Research Needs

This review examines the current techniques for synthesizing silver nanoparticles. It has been determined that over the last ten years, numerous efforts have been attempted to achieve a biological nanoparticle synthesis. This approach offers several advantages over chemical and physical methods since it is less expensive, less harmful to the environment, and scaled up quickly for massive production scale. Due to a growing interest in green chemistry and the desire to develop sustainable methods for producing metal nanoparticles, particularly silver nanoparticles, there has been a rise in the utilization of green synthesis routes. While many studies have employed extract of the plant as stabilizing or protecting agents for nanoparticle production, there have been a few investigations into the use of bioflocculant for the synthesis of nanoparticles. The current review focuses on the synthesis, characterization, and application of silver nanoparticles, with a particular emphasis on their synthesis employing micro-organism by-products, and their use in wastewater treatment and dye removal. In the past ten years, significant advancements have been made in the field of using marine microorganisms to produce metallic nanoparticles for biological and biomedical purposes. However, there is still a lot of work needed to create a green synthesis process that can control particle size and shape. The poor speed of this process makes managing the size, shape, and consistency of nanoparticles a considerable problem, which might be improved by shortening the synthesis time. Currently, there is limited research on the green synthesis of metallic nanoparticles using bioflocculants, and more studies on extracellular biosynthesis are necessary.

Despite this, the biosynthesis process and its industrial applications are still at the laboratory stage, and more efforts are required to scale up production. Although there are many established techniques for creating nanoparticles, it is still necessary to optimize and develop new approaches to enhance their yield, size, and shape. Understanding the properties and behavior of nanoparticles is essential, and new approaches are required to characterize them in different environments, including *in vivo* and in real-time, to investigate their interactions with biological systems. The potential application of silver nanoparticles is vast and varied, and further research should aim to develop innovative uses in domains such as health care, electricity, and the remediation of environments. However, as with any emerging technology, it is crucial to thoroughly investigate the environmental impact of nanoparticles to avoid unintended consequences. Future research should therefore focus on analyzing nanoparticles’ environmental impact, including their persistence in the environment, ability to aggregate in living creatures, and consequences on ecosystems. More studies are required for better comprehension of the mechanisms of AgNP cytotoxicity.

## Figures and Tables

**Table 1:** Some bacteria strains were reported in the synthesis of AgNPs.

Nanometal	Bacteria	Size (nm)	Shape	Citation
AgNPs	*Bacillus siamensis*	25–50	Spherical	[113]
	*Klebsiella pneumonia*	26.84–44.42	Spherical	[114]
	*Bacillus subtilis*	3–20	Spherical	[115]
	*Lactobacillus bulgaricus*	30.65–100	Spherical	[116]
	*Bacillus cereus*	45.4 and 90.8	Spherical and polygon	[117]

**Table 2:** Factors affecting biosynthesis of silver nanoparticles.

NPs	Bacteria	Optimized Parameters	Optimized Conditions	Nanoparticles Characteristics	Citation
AgNPs	*Bacillus* ROM6	Temperature	35 °C	25 nm, spherical	[154]
	*Bacillus cereus*	Concentration, inoculum size, temperature, time, and pH	AgNO_3_ (1 mM), 8.7 mL, 48.5 °C, 69 h, pH 9	5 to 7.06 nm, spherical	[155]
	*Kocuria rhizophila* BR-1	Temperature, concentration, pH, and time	54.07 °C, 1.17 mM, 9.89 pH, 13.24 h	10–200 nm, spherical	[156]
	*Lactobacillus bulgaricus*	Temperature and concentration	30 °C, AgNO_3_ (4 mM)	30–100 nm, spherical	[116]
	*Lactobacillus spicheri* G2 (JX481912)	Temperature and pH	37 °C, pH 7	20 nm, spherical/square in shape	[157]
	*Spirulina platensis*	Temperature and pH	50 °C, pH 10	2.23–14.68 nm, spherical	[158]

## Data Availability

None applicable.

## Abbreviations

AgNO_3_	silver nitrate
AgNPs	silver nanoparticles
BOD	Biological Oxygen demand
COD	Chemical Oxygen Demand
DLS	Dynamic light scattering
ERA	Epixide ricinoleic acid
FTIR	Fourier transform infrared
MIC	Minimum Inhibitory Concentration
Min	minutes
NPs	nanoparticles
RA	ricinoleic acid
SEM	Scanning electron microscopy
SPR	Surface Plasmon Resonance
TEM	Transmission electron microscopy
USFDA	United States Food and Drug Administration
USEPA	United States Environmental Protection Agency
XRD	X-ray diffraction

## References

[1] Chakkaravarthy D.N., Balakrishnan T. (2019). Water scarcity-challenging the future. Int. J. Agric. Environ. Biotechnol..

[2] Enitan-Folami A., Lanrewaju A., Swalaha F. (2022). Inactivation of Waterborne Pathogens Using Medicinal Plants.

[3] Alashti H.A., Bozorg-Haddad O., Bocchiola D. (2021). Passive defense in water resources. Water Resources: Future Perspectives, Challenges, Concepts and Necessities.

[4] Ali Z., Ahmad R. (2020). Nanotechnology for water treatment. Environmental Nanotechnology Volume 3.

[5] Das N., Ojha N., Mandal S.K. (2021). Wastewater treatment using plant-derived bioflocculants: Green chemistry approach for safe environment. Water Sci. Technol..

[6] Ngema S., Basson A., Maliehe T. (2020). Synthesis, characterization and application of polyacrylamide grafted bioflocculant. Phys. Chem. Earth Parts A/B/C.

[7] Soren S., Panda P., Chakroborty S. (2023). Nanotechnology in Water and Wastewater Treatment. Agricultural and Environmental Nanotechnology: Novel Technologies and their Ecological Impact.

[8] Elgarahy A.M., Elwakeel K.Z., Akhdhar A., Hamza M.F. (2021). Recent advances in greenly synthesized nanoengineered materials for water/wastewater remediation: An overview. Nanotechnol. Environ. Eng..

[9] Afre R.A. (2020). Nanotechnology Industry: Scenario of Intellectual Property Rights. Intellectual Property Issues in Nanotechnology.

[10] Ntombela Z.G., Pullabhotla V.S.R., Basson A.K. (2022). Biosafety, Optimization, and Application of Bioflocculant-Synthesized Zinc Oxide Nanoparticles. BioNanoScience.

[11] Srivastava S., Bhargava A., Srivastava S., Bhargava A. (2022). Green nanotechnology: An overview. Green Nanoparticles: The Future of Nanobiotechnology.

[12] Zain M., Yasmeen H., Yadav S.S., Amir S., Bilal M., Shahid A., Khurshid M. (2022). Applications of nanotechnology in biological systems and medicine. Nanotechnology for Hematology, Blood Transfusion, and Artificial Blood.

[13] Kianfar E. (2021). Magnetic nanoparticles in targeted drug delivery: A review. J. Supercond. Nov. Magn..

[14] Archana P., Janarthanan B., Bhuvana S., Rajiv P., Sharmila S. (2022). Concert of zinc oxide nanoparticles synthesized using Cucumis melo by green synthesis and the antibacterial activity on pathogenic bacteria. Inorg. Chem. Commun..

[15] Zhao Z., Li M., Zeng J., Huo L., Liu K., Wei R., Ni K., Gao J. (2022). Recent advances in engineering iron oxide nanoparticles for effective magnetic resonance imaging. Bioact. Mater..

[16] Thakur P., Bhardwaj K.K., Gupta R. (2018). Nanotechnology and Nanomaterials: Applications and Environmental Issues. Green Sustain. Adv. Mater. Appl..

[17] Kaabipour S., Hemmati S. (2021). A review on the green and sustainable synthesis of silver nanoparticles and one-dimensional silver nanostructures. Beilstein J. Nanotechnol..

[18] Aboyewa J.A., Sibuyi N.R., Meyer M., Oguntibeju O.O. (2021). Green synthesis of metallic nanoparticles using some selected medicinal plants from southern africa and their biological applications. Plants.

[19] Bahrulolum H., Nooraei S., Javanshir N., Tarrahimofrad H., Mirbagheri V.S., Easton A.J., Ahmadian G. (2021). Green synthesis of metal nanoparticles using microorganisms and their application in the agrifood sector. J. Nanobiotechnol..

[20] Shafey A.M.E. (2020). Green synthesis of metal and metal oxide nanoparticles from plant leaf extracts and their applications: A review. Green Process. Synth..

[21] Yazdanian M., Rostamzadeh P., Rahbar M., Alam M., Abbasi K., Tahmasebi E., Tebyaniyan H., Ranjbar R., Seifalian A., Yazdanian A. (2022). The potential application of green-synthesized metal nanoparticles in dentistry: A comprehensive review. Bioinorg. Chem. Appl..

[22] Baig N., Kammakakam I., Falath W. (2021). Nanomaterials: A review of synthesis methods, properties, recent progress, and challenges. Mater. Adv..

[23] Galatage S.T., Hebalkar A.S., Dhobale S.V., Mali O.R., Kumbhar P.S., Nikade S.V., Killedar S.G. (2021). Silver nanoparticles: Properties, synthesis, characterization, applications and future trends. Silver Micro-Nanoparticles-Properties, Synthesis, Characterization, and Applications.

[24] Coetzee D., Venkataraman M., Militky J., Petru M. (2020). Influence of nanoparticles on thermal and electrical conductivity of composites. Polymers.

[25] Bruna T., Maldonado-Bravo F., Jara P., Caro N. (2021). Silver nanoparticles and their antibacterial applications. Int. J. Mol. Sci..

[26] Mughal B., Zaidi S.Z.J., Zhang X., Hassan S.U. (2021). Biogenic nanoparticles: Synthesis, characterisation and applications. Appl. Sci..

[27] Yin I.X., Zhang J., Zhao I.S., Mei M.L., Li Q., Chu C.H. (2020). The antibacterial mechanism of silver nanoparticles and its application in dentistry. Int. J. Nanomed..

[28] Salem S.S., Fouda A. (2021). Green synthesis of metallic nanoparticles and their prospective biotechnological applications: An overview. Biol. Trace Elem. Res..

[29] Flores-Rojas G.G., López-Saucedo F., Bucio E., Inamuddin, Alrooqi A., Altalhi T. (2021). Chapter 10—Green synthesized zinc oxide nanomaterials and its therapeutic applications. Green Sustainable Process for Chemical and Environmental Engineering and Science.

[30] Naseem T., Durrani T. (2021). The role of some important metal oxide nanoparticles for wastewater and antibacterial applications: A review. Environ. Chem. Ecotoxicol..

[31] Manojkumar U., Kaliannan D., Srinivasan V., Balasubramanian B., Kamyab H., Mussa Z.H., Palaniyappan J., Mesbah M., Chelliapan S., Palaninaicker S. (2023). Green synthesis of zinc oxide nanoparticles using Brassica oleracea var. botrytis leaf extract: Photocatalytic, antimicrobial and larvicidal activity. Chemosphere.

[32] Atri A., Echabaane M., Bouzidi A., Harabi I., Soucase B.M., Chaâbane R.B. (2023). Green synthesis of copper oxide nanoparticles using Ephedra Alata plant extract and a study of their antifungal, antibacterial activity and photocatalytic performance under sunlight. Heliyon.

[33] Derakhshani E., Asri M., Naghizadeh A. (2023). Plant-Based Green Synthesis of Copper Oxide Nanoparticles Using Berberis vulgaris Leaf Extract: An Update on Their Applications in Antibacterial Activity. BioNanoScience.

[34] Hussain R., Zafar A., Hasan M., Tariq T., Saif M.S., Waqas M., Tariq F., Anum M., Anjum S.I., Shu X. (2023). Casting zinc oxide nanoparticles using Fagonia blend microbial arrest. Appl. Biochem. Biotechnol..

[35] Dappula S.S., Kandrakonda Y.R., Shaik J.B., Mothukuru S.L., Lebaka V.R., Mannarapu M., Amooru G.D. (2023). Biosynthesis of zinc oxide nanoparticles using aqueous extract of Andrographis alata: Characterization, optimization and assessment of their antibacterial, antioxidant, antidiabetic and anti-Alzheimer’s properties. J. Mol. Struct..

[36] Govindarajan D.K., Selvaraj V., Selvaraj A.S.J.M., Hameed S.S., Pandiarajan J., Veluswamy A. (2023). Green synthesis of silver micro-and nano-particles using phytochemical extracts of Cymbopogon citratus exhibits antibacterial properties. Mater. Today: Proc..

[37] Braim F.S., Ab Razak N.N.A.N., Aziz A.A., Dheyab M.A., Ismael L.Q. (2023). Rapid green-assisted synthesis and functionalization of superparamagnetic magnetite nanoparticles using Sumac extract and assessment of their cellular toxicity, uptake, and anti-metastasis property. Ceram. Int..

[38] Ihsan M., Din I.U., Alam K., Munir I., Mohamed H.I., Khan F. (2023). Green Fabrication, Characterization of Zinc Oxide Nanoparticles Using Plant Extract of *Momordica charantia* and *Curcuma zedoaria* and Their Antibacterial and Antioxidant Activities. Appl. Biochem. Biotechnol..

[39] Arun S., Karthik B., Yatish K., Prashanth K., Balakrishna G.R. (2023). Green synthesis of copper oxide nanoparticles using the Bombax ceiba plant: Biodiesel production and nano-additive to investigate diesel engine performance-emission characteristics. Energy.

[40] Sampath G., Govarthanan M., Rameshkumar N., Vo D.-V.N., Krishnan M., Sivasankar P., Kayalvizhi N. (2023). Eco-friendly biosynthesis metallic silver nanoparticles using Aegle marmelos (Indian bael) and its clinical and environmental applications. Appl. Nanosci..

[41] Dlamini N.G., Basson A.K., Pullabhotla V. (2019). Biosynthesis and characterization of copper nanoparticles using a bioflocculant extracted from Alcaligenis faecalis HCB2. Adv. Sci. Eng. Med..

[42] Adebayo-Tayo B.C., Adeleke R.O., Adekanmbi A.O. (2022). Biogenic Silver and Magnetic Nanoparticles Using Bacillus subtilis B2 Bioflocculants; Production, Properties and Antibacterial Potential in Dairy Wastewater Treatment. Chem. Afr..

[43] Tsilo P.H., Basson A.K., Ntombela Z.G., Dlamini N.G., Pullabhotla R.V. (2023). Application of Iron Nanoparticles Synthesized from a Bioflocculant Produced by Yeast Strain *Pichia kudriavzevii* Obtained from Kombucha Tea SCOBY in the Treatment of Wastewater. Int. J. Mol. Sci..

[44] Jiang Z., Li L., Huang H., He W., Ming W. (2022). Progress in Laser Ablation and Biological Synthesis Processes:“Top-Down” and “Bottom-Up” Approaches for the Green Synthesis of Au/Ag Nanoparticles. Int. J. Mol. Sci..

[45] Yuda A.P., Koraag P.Y.E., Iskandar F., Wasisto H.S., Sumboja A. (2021). Advances of the top-down synthesis approach for high-performance silicon anodes in Li-ion batteries. J. Mater. Chem. A.

[46] Krishnia L., Thakur P., Thakur A. (2022). Synthesis of nanoparticles by physical route. Synthesis and Applications of Nanoparticles.

[47] Amrute A.P., De Bellis J., Felderhoff M., Schüth F. (2021). Mechanochemical synthesis of catalytic materials. Chem.-A Eur. J..

[48] Mehta K., Baruah P.K. (2022). A comprehensive review and outlook on the experimental techniques to investigate the complex dynamics of pulsed laser ablation in liquid for nanoparticle synthesis. Rev. Sci. Instrum..

[49] Zia A.W., Birkett M., Badshah M.A., Iqbal M. (2021). Progress in-situ synthesis of graphitic carbon nanoparticles with physical vapour deposition. Prog. Cryst. Growth Charact. Mater..

[50] Lee S.H., Jun B.-H. (2019). Silver nanoparticles: Synthesis and application for nanomedicine. Int. J. Mol. Sci..

[51] Patel R.R., Singh S.K., Singh M. (2023). Green synthesis of silver nanoparticles: Methods, biological applications, delivery and toxicity. Mater. Adv..

[52] Karunakaran R.G., Dhamodharan R. (2020). 2 Tailor-made polymer–nanohybrid materials via reversible deactivation radical polymerization (RDRP). Reversible Deactivation Radical Polymerization: Synthesis and Applications of Functional Polymers.

[53] Abid N., Khan A.M., Shujait S., Chaudhary K., Ikram M., Imran M., Haider J., Khan M., Khan Q., Maqbool M. (2022). Synthesis of nanomaterials using various top-down and bottom-up approaches, influencing factors, advantages, and disadvantages: A review. Adv. Colloid Interface Sci..

[54] Gundo S., Parauha Y.R., Singh N., Dhoble S. (2021). Eco-friendly synthesis route of silver nanoparticle: A review. J. Phys. Conf. Ser..

[55] Singh P., Kim Y.-J., Zhang D., Yang D.-C. (2016). Biological synthesis of nanoparticles from plants and microorganisms. Trends Biotechnol..

[56] Natsuki J., Natsuki T., Hashimoto Y. (2015). A review of silver nanoparticles: Synthesis methods, properties and applications. Int. J. Mater. Sci. Appl..

[57] Singh A., Gautam P.K., Verma A., Singh V., Shivapriya P.M., Shivalkar S., Sahoo A.K., Samanta S.K. (2020). Green synthesis of metallic nanoparticles as effective alternatives to treat antibiotics resistant bacterial infections: A review. Biotechnol. Rep..

[58] Srivastava S., Usmani Z., Atanasov A.G., Singh V.K., Singh N.P., Abdel-Azeem A.M., Prasad R., Gupta G., Sharma M., Bhargava A. (2021). Biological nanofactories: Using living forms for metal nanoparticle synthesis. Mini Rev. Med. Chem..

[59] Aswathi V., Meera S., Maria C.A., Nidhin M. (2022). Green synthesis of nanoparticles from biodegradable waste extracts and their applications: A critical review. Nanotechnol. Environ. Eng..

[60] Paul A., Roychoudhury A. (2021). Go green to protect plants: Repurposing the antimicrobial activity of biosynthesized silver nanoparticles to combat phytopathogens. Nanotechnol. Environ. Eng..

[61] Patil T., Gambhir R., Vibhute A., Tiwari A.P. (2023). Gold nanoparticles: Synthesis methods, functionalization and biological applications. J. Clust. Sci..

[62] Sampath G., Chen Y.-Y., Rameshkumar N., Krishnan M., Nagarajan K., Shyu D.J. (2022). Biologically synthesized silver nanoparticles and their diverse applications. Nanomaterials.

[63] Usmanac A.I., Aziza A.A., Noqtab O.A. (2019). Application of green synthesis of gold nanoparticles: A review. J. Teknol..

[64] Alharbi N.S., Alsubhi N.S., Felimban A.I. (2022). Green synthesis of silver nanoparticles using medicinal plants: Characterization and application. J. Radiat. Res. Appl. Sci..

[65] Chatterjee A., Kwatra N., Abraham J. (2020). Nanoparticles fabrication by plant extracts. Phytonanotechnology.

[66] Zuecco G., Amin A., Frentress J., Engel M., Marchina C., Anfodillo T., Borga M., Carraro V., Scandellari F., Tagliavini M. (2020). A comparative study of plant water extraction methods for isotopic analyses: Scholander-type pressure chamber vs. cryogenic vacuum distillation. Hydrol. Earth Syst. Sci. Discuss..

[67] Khan S., Singh S., Gaikwad S., Nawani N., Junnarkar M., Pawar S.V. (2020). Optimization of process parameters for the synthesis of silver nanoparticles from Piper betle leaf aqueous extract, and evaluation of their antiphytofungal activity. Environ. Sci. Pollut. Res..

[68] Liu Y., Kang S., Li K., Chen J., Bae B., Hwang I., Ahn E.-Y., Park Y., Chun K.-H., Lee J. (2022). Ecofriendly and enhanced biogenic synthesis of silver nanoparticles using deep eutectic solvent-based green tea extracts. J. Clean. Prod..

[69] Soni V., Raizada P., Singh P., Cuong H.N., Rangabhashiyam S., Saini A., Saini R.V., Van Le Q., Nadda A.K., Le T.-T. (2021). Sustainable and green trends in using plant extracts for the synthesis of biogenic metal nanoparticles toward environmental and pharmaceutical advances: A review. Environ. Res..

[70] Haji B.S., Barzinjy A.A. (2022). Citrullus Colocynthis Fruit Extract Mediated Green Synthesis of Silver Nanoparticles: The Impact of pH, Temperature, and Silver Nitrate Concentration. e-J. Surf. Sci. Nanotechnol..

[71] Marulasiddeshwara M., Dakshayani S., Kumar M.S., Chethana R., Kumar P.R., Devaraja S. (2017). Facile-one pot-green synthesis, antibacterial, antifungal, antioxidant and antiplatelet activities of lignin capped silver nanoparticles: A promising therapeutic agent. Mater. Sci. Eng. C.

[72] Chakrabartty I. (2022). Plant-Based Nanoparticles and Their Applications. Diverse Applications of Nanotechnology in the Biological Sciences.

[73] Phull A.-R., Ali A., Ali A., Abbasi S., Zia M., Khaskheli M.H., Kamal M.A. (2020). Synthesis of Silver Nanoparticles using Euphorbia wallichii Extract and Assessment of their Bio-functionalities. Med. Chem..

[74] Gardea-Torresdey J.L., Gomez E., Peralta-Videa J.R., Parsons J.G., Troiani H., Jose-Yacaman M. (2003). Alfalfa sprouts: A natural source for the synthesis of silver nanoparticles. Langmuir.

[75] Jini D., Sharmila S. (2020). Green synthesis of silver nanoparticles from Allium cepa and its in vitro antidiabetic activity. Mater. Today Proc..

[76] Hekmati M., Hasanirad S., Khaledi A., Esmaeili D. (2020). Green synthesis of silver nanoparticles using extracts of Allium rotundum l, Falcaria vulgaris Bernh, and Ferulago angulate Boiss, and their antimicrobial effects in vitro. Gene Rep..

[77] Viana A.D., Nobrega E.T., Moraes E.P., Neto A.O.W., Menezes F.G., Gasparotto L.H. (2020). Castor oil derivatives in the environmentally friendly one-pot synthesis of silver nanoparticles: Application in cysteine sensing. Mater. Res. Bull..

[78] Singh R., Hano C., Tavanti F., Sharma B. (2021). Biogenic Synthesis and Characterization of Antioxidant and Antimicrobial Silver Nanoparticles Using Flower Extract of Couroupita guianensis Aubl. Materials.

[79] Verma A., Preet S. (2022). Novel Green Synthesis of Combinational Silver Nanoparticles using Jatropha integerrima for Dengue Vector Control and Cytotoxicity Assessment. Proc. Natl. Acad. Sci. USA India Sect. B Biol. Sci..

[80] Nahar K., Yang D.-C., Rupa E.J., Khatun M., Al-Reza S.M. (2020). Eco-friendly synthesis of silver nanoparticles from Clerodendrum viscosum leaf extract and its antibacterial potential. Nanomed. Res. J..

[81] Huq M.A., Ashrafudoulla M., Rahman M.M., Balusamy S.R., Akter S. (2022). Green synthesis and potential antibacterial applications of bioactive silver nanoparticles: A review. Polymers.

[82] Arif R., Uddin R. (2021). A review on recent developments in the biosynthesis of silver nanoparticles and its biomedical applications. Med. Devices Sens..

[83] Win T.T., Khan S., Fu P. (2020). Fungus-(*Alternaria* sp.) mediated silver nanoparticles synthesis, characterization, and screening of antifungal activity against some phytopathogens. J. Nanotechnol..

[84] Talie M., Wani A.H., Ahmad N., Bhat M.Y., War J.M. (2020). Green synthesis of silver nanoparticles (AgNPs) using Helvella leucopus Pers. and their antimycotic activity against fungi causing fungal rot of Apple. Asian J. Pharm. Clin. Res..

[85] Rehman S., Farooq R., Jermy R., Mousa Asiri S., Ravinayagam V., Al Jindan R., Alsalem Z., Shah M.A., Reshi Z., Sabit H. (2020). A wild fomes fomentarius for biomediation of one pot synthesis of titanium oxide and silver nanoparticles for antibacterial and anticancer application. Biomolecules.

[86] Rehman S., Jermy R., Asiri S.M., Shah M.A., Farooq R., Ravinayagam V., Ansari M.A., Alsalem Z., Al Jindan R., Reshi Z. (2020). Using Fomitopsis pinicola for bioinspired synthesis of titanium dioxide and silver nanoparticles, targeting biomedical applications. RSC Adv..

[87] Danagoudar A., Pratap G., Shantaram M., Ghosh K., Kanade S.R., Joshi C.G. (2020). Characterization, cytotoxic and antioxidant potential of silver nanoparticles biosynthesised using endophytic fungus (*Penicillium citrinum* CGJ-C1). Mater. Today Commun..

[88] Owaid M.N., Rabeea M.A., Aziz A.A., Jameel M.S., Dheyab M.A. (2022). Mycogenic fabrication of silver nanoparticles using Picoa, Pezizales, characterization and their antifungal activity. Environ. Nanotechnol. Monit. Manag..

[89] Gupta P., Rai N., Verma A., Saikia D., Singh S.P., Kumar R., Singh S.K., Kumar D., Gautam V. (2022). Green-Based Approach to Synthesize Silver Nanoparticles Using the Fungal Endophyte *Penicillium oxalicum* and Their Antimicrobial, Antioxidant, and In Vitro Anticancer Potential. ACS Omega.

[90] Joy F., Devasia J., Nizam A., Lakshmaiah V.V., Krishna S.B.N. (2023). Fungi-templated silver nanoparticle composite: Synthesis, characterization, and its applications. Appl. Sci..

[91] Yadav S., Sharma A.K., Kumar P. (2020). Nanoscale self-assembly for therapeutic delivery. Front. Bioeng. Biotechnol..

[92] Burdușel A.-C., Gherasim O., Grumezescu A.M., Mogoantă L., Ficai A., Andronescu E. (2018). Biomedical applications of silver nanoparticles: An up-to-date overview. Nanomaterials.

[93] El-Moslamy S.H., Elkady M.F., Rezk A.H., Abdel-Fattah Y.R. (2017). Applying Taguchi design and large-scale strategy for mycosynthesis of nano-silver from endophytic *Trichoderma harzianum* SYA. F4 and its application against phytopathogens. Sci. Rep..

[94] Ammar H.A., El Aty A.A.A., El Awdan S.A. (2021). Extracellular myco-synthesis of nano-silver using the fermentable yeasts *Pichia kudriavzevii* HA-NY2 and *Saccharomyces uvarum* HA-NY3, and their effective biomedical applications. Bioprocess Biosyst. Eng..

[95] Awad M.A., Eid A.M., Elsheikh T.M., Al-Faifi Z.E., Saad N., Sultan M.H., Selim S., Al-Khalaf A.A., Fouda A. (2022). Mycosynthesis, characterization, and mosquitocidal activity of silver nanoparticles fabricated by *Aspergillus niger* strain. J. Fungi.

[96] Dutta T., Chattopadhyay A.P., Ghosh N.N., Khatua S., Acharya K., Kundu S., Mitra D., Das M. (2020). Biogenic silver nanoparticle synthesis and stabilization for apoptotic activity; insights from experimental and theoretical studies. Chem. Pap..

[97] Rozhin A., Batasheva S., Kruychkova M., Cherednichenko Y., Rozhina E., Fakhrullin R. (2021). Biogenic silver nanoparticles: Synthesis and application as antibacterial and antifungal agents. Micromachines.

[98] Trzcińska-Wencel J., Wypij M., Rai M., Golińska P. (2023). Biogenic nanosilver bearing antimicrobial and antibiofilm activities and its potential for application in agriculture and industry. Front. Microbiol..

[99] Wang D., Xue B., Wang L., Zhang Y., Liu L., Zhou Y. (2021). Fungus-mediated green synthesis of nano-silver using Aspergillus sydowii and its antifungal/antiproliferative activities. Sci. Rep..

[100] Alves M.F., Murray P.G. (2022). Biological Synthesis of Monodisperse Uniform-Size Silver Nanoparticles (AgNPs) by Fungal Cell-Free Extracts at Elevated Temperature and pH. J. Fungi.

[101] Gudikandula K., Vadapally P., Singara Charya M.A. (2017). Biogenic synthesis of silver nanoparticles from white rot fungi: Their characterization and antibacterial studies. OpenNano.

[102] Hu X., Saravanakumar K., Jin T., Wang M.-H. (2019). Mycosynthesis, characterization, anticancer and antibacterial activity of silver nanoparticles from endophytic fungus *Talaromyces purpureogenus*. Int. J. Nanomed..

[103] Kathiraven T., Sundaramanickam A., Shanmugam N., Balasubramanian T. (2015). Green synthesis of silver nanoparticles using marine algae Caulerpa racemosa and their antibacterial activity against some human pathogens. Appl. Nanosci..

[104] El-Rafie H., El-Rafie M., Zahran M. (2013). Green synthesis of silver nanoparticles using polysaccharides extracted from marine macro algae. Carbohydr. Polym..

[105] Öztürk B.Y., Gürsu B.Y., Dağ İ. (2020). Antibiofilm and antimicrobial activities of green synthesized silver nanoparticles using marine red algae *Gelidium corneum*. Process Biochem..

[106] Fatima R., Priya M., Indurthi L., Radhakrishnan V., Sudhakaran R. (2020). Biosynthesis of silver nanoparticles using red algae *Portieria hornemannii* and its antibacterial activity against fish pathogens. Microb. Pathog..

[107] Huq M.A. (2020). Green synthesis of silver nanoparticles using *Pseudoduganella eburnea* MAHUQ-39 and their antimicrobial mechanisms investigation against drug resistant human pathogens. Int. J. Mol. Sci..

[108] Agarwal A., Pathera A.K., Kaushik R., Kumar N., Dhull S.B., Arora S., Chawla P. (2020). Succinylation of milk proteins: Influence on micronutrient binding and functional indices. Trends Food Sci. Technol..

[109] Lule V., Tomar S.K., Sequeira S. (2020). Metal Nanoparticles of Microbial Origin and Their Antimicrobial Applications in Food Industries. Nanotechnological Approaches in Food Microbiology.

[110] Ali I., Qiang T.Y., Ilahi N., Adnan M., Sajjad W. (2018). Green synthesis of silver nanoparticles by using bacterial extract and its antimicrobial activity against pathogens. Int. J. Biosci..

[111] Bharti S., Mukherji S., Mukherji S. (2020). Extracellular synthesis of silver nanoparticles by *Thiosphaera pantotropha* and evaluation of their antibacterial and cytotoxic effects. 3 Biotech.

[112] Otari S., Patil R., Ghosh S., Thorat N., Pawar S. (2015). Intracellular synthesis of silver nanoparticle by actinobacteria and its antimicrobial activity. Spectrochim. Acta Part A Mol. Biomol. Spectrosc..

[113] Ibrahim E., Fouad H., Zhang M., Zhang Y., Qiu W., Yan C., Li B., Mo J., Chen J. (2019). Biosynthesis of silver nanoparticles using endophytic bacteria and their role in inhibition of rice pathogenic bacteria and plant growth promotion. RSC Adv..

[114] Saleh M.N., Alwan S.K. (2020). Bio-synthesis of silver nanoparticles from bacteria Klebsiella pneumonia: Their characterization and antibacterial studies. J. Phys. Conf. Ser..

[115] Alsamhary K.I. (2020). Eco-friendly synthesis of silver nanoparticles by Bacillus subtilis and their antibacterial activity. Saudi J. Biol. Sci..

[116] Naseer Q., Xue X., Wang X., Dang S., Din S., Jamil J. (2021). Synthesis of silver nanoparticles using Lactobacillus bulgaricus and assessment of their antibacterial potential. Braz. J. Biol..

[117] Alfryyan N., Kordy M.G., Abdel-Gabbar M., Soliman H.A., Shaban M. (2022). Characterization of the biosynthesized intracellular and extracellular plasmonic silver nanoparticles using Bacillus cereus and their catalytic reduction of methylene blue. Sci. Rep..

[118] Shahadat M., Teng T.T., Rafatullah M., Shaikh Z., Sreekrishnan T., Ali S.W. (2017). Bacterial bioflocculants: A review of recent advances and perspectives. Chem. Eng. J..

[119] Dlamini N.G., Basson A.K., Pullabhotla R.V. (2020). Wastewater treatment by a polymeric bioflocculant and iron nanoparticles synthesized from a bioflocculant. Polymers.

[120] Vimala R., Escaline J.L., Sivaramakrishnan S. (2020). Characterization of self-assembled bioflocculant from the microbial consortium and its applications. J. Environ. Manag..

[121] Law J.W.-F., Pusparajah P., Ab Mutalib N.-S., Wong S.H., Goh B.-H., Lee L.-H. (2019). A review on mangrove actinobacterial diversity: The roles of *Streptomyces* and novel species discovery. Prog. Microbes Mol. Biol..

[122] Nangare S.N., Patil P.O. (2020). Green Synthesis of Silver Nanoparticles: An Eco-Friendly Approach. Nano Biomed. Eng..

[123] Adebayo E., Kumi E., Ajibade V., Akinjo A., Abiola O., Raji M., Adepoju Y., Oduoye O. (2020). Biosynthesis and bactericidal activity of silver nanoparticles (AgNPs) using cell-free bioflocculant from domestic wastewater bacterial consortium. IOP Conf. Ser. Mater. Sci. Eng..

[124] Hassan A.M., Hegazy G.E., Elela G.M.A., El Naby H.M.A., Dusuki Y.Y.M., Ibrahim H.A. (2021). Bioflocculant production by marine psychrotolerant Psychrobacter cibarius H41A KF207755 with its special role in silver nanoparticles production. Egypt. J. Aquat. Biol. Fish.

[125] Ajani T.F., Adebayo-Tayo B.C., Omoniyi S. (2021). Bio-fabrication, Optimization and Characterization of Silver Nanoparticles Using *Streptomyces* sp. HDW7 and *Nocardia* sp. OX5 Bioflocculants; Flocculation and Antibacterial Efficiency. Acta Microbiol. Bulg..

[126] Muthulakshmi L., Rajini N., Rajalu A.V., Siengchin S., Kathiresan T. (2017). Synthesis and characterization of cellulose/silver nanocomposites from bioflocculant reducing agent. Int. J. Biol. Macromol..

[127] Sajayan A., Kiran G.S., Priyadharshini S., Poulose N., Selvin J. (2017). Revealing the ability of a novel polysaccharide bioflocculant in bioremediation of heavy metals sensed in a *Vibrio bioluminescence* reporter assay. Environ. Pollut..

[128] Zeidman A.B., Rodriguez-Narvaez O.M., Moon J., Bandala E.R. (2020). Removal of antibiotics in aqueous phase using silica-based immobilized nanomaterials: A review. Environ. Technol. Innov..

[129] Shamzhy M., Opanasenko M., Concepción P., Martínez A. (2019). New trends in tailoring active sites in zeolite-based catalysts. Chem. Soc. Rev..

[130] Skiba M., Vorobyova V. (2022). Green synthesis and characterization of silver nanoparticles using *Prunus persica* L.(peach pomace) with natural deep eutectic solvent and plasma-liquid process. Chem. Pap..

[131] Dikshit P.K., Kumar J., Das A.K., Sadhu S., Sharma S., Singh S., Gupta P.K., Kim B.S. (2021). Green synthesis of metallic nanoparticles: Applications and limitations. Catalysts.

[132] Al-Kordy H.M., Sabry S.A., Mabrouk M.E. (2021). Statistical optimization of experimental parameters for extracellular synthesis of zinc oxide nanoparticles by a novel haloalaliphilic *Alkalibacillus* sp. W7. Sci. Rep..

[133] Balavandy S.K., Shameli K., Biak D.R., Abidin Z.Z. (2014). Stirring time effect of silver nanoparticles prepared in glutathione mediated by green method. Chem. Cent. J..

[134] Khan I., Saeed K., Khan I. (2019). Nanoparticles: Properties, applications and toxicities. Arab. J. Chem..

[135] Izak-Nau E., Huk A., Reidy B., Uggerud H., Vadset M., Eiden S., Voetz M., Himly M., Duschl A., Dusinska M. (2015). Impact of storage conditions and storage time on silver nanoparticles’ physicochemical properties and implications for their biological effects. Rsc Adv..

[136] Fu L.-M., Hsu J.-H., Shih M.-K., Hsieh C.-W., Ju W.-J., Chen Y.-W., Lee B.-H., Hou C.-Y. (2021). Process optimization of silver nanoparticle synthesis and its application in mercury detection. Micromachines.

[137] Fernando I., Zhou Y. (2019). Impact of pH on the stability, dissolution and aggregation kinetics of silver nanoparticles. Chemosphere.

[138] Iqtedar M., Aslam M., Akhyar M., Shehzaad A., Abdullah R., Kaleem A. (2019). Extracellular biosynthesis, characterization, optimization of silver nanoparticles (AgNPs) using *Bacillus mojavensis* BTCB15 and its antimicrobial activity against multidrug resistant pathogens. Prep. Biochem. Biotechnol..

[139] Elsayed M.A., Othman A.M., Hassan M.M., Elshafei A.M. (2018). Optimization of silver nanoparticles biosynthesis mediated by *Aspergillus niger* NRC1731 through application of statistical methods: Enhancement and characterization. 3 Biotech.

[140] Patra J.K., Baek K.-H. (2015). Green nanobiotechnology: Factors affecting synthesis and characterization techniques. J. Nanomater..

[141] Lashari A., Hassan S.M., Mughal S.S. (2022). Biosynthesis, Characterization and Biological Applications of BaO Nanoparticles using *Linum usitatissimum*. Am. J. Appl. Sci. Res..

[142] Stankic S., Suman S., Haque F., Vidic J. (2016). Pure and multi metal oxide nanoparticles: Synthesis, antibacterial and cytotoxic properties. J. Nanobiotechnol..

[143] Monteiro R.A., Camara M.C., de Oliveira J.L., Campos E.V.R., Carvalho L.B., de Freitas Proenca P.L., Guilger-Casagrande M., Lima R., do Nascimento J., Gonçalves K.C. (2021). Zein based-nanoparticles loaded botanical pesticides in pest control: An enzyme stimuli-responsive approach aiming sustainable agriculture. J. Hazard. Mater..

[144] Saeed S., Iqbal A., Ashraf M.A. (2020). Bacterial-mediated synthesis of silver nanoparticles and their significant effect against pathogens. Environ. Sci. Pollut. Res..

[145] Restrepo C.V., Villa C.C. (2021). Synthesis of silver nanoparticles, influence of capping agents, and dependence on size and shape: A review. Environ. Nanotechnol. Monit. Manag..

[146] Liu H., Zhang H., Wang J., Wei J. (2020). Effect of temperature on the size of biosynthesized silver nanoparticle: Deep insight into microscopic kinetics analysis. Arab. J. Chem..

[147] Vivekanandhan P., Deepa S., Kweka E.J., Shivakumar M.S. (2018). Toxicity of Fusarium oxysporum-VKFO-01 derived silver nanoparticles as potential inseciticide against three mosquito vector species (Diptera: Culicidae). J. Clust. Sci..

[148] Naser H., Alghoul M., Hossain M.K., Asim N., Abdullah M., Ali M.S., Alzubi F.G., Amin N. (2019). The role of laser ablation technique parameters in synthesis of nanoparticles from different target types. J. Nanoparticle Res..

[149] Zikmund T., Bulíř J., Novotný M., Fekete L., Chertopalov S., Irimiciuc S.A., Klementová M., Balogová J., Lančok J. (2023). Silver Nanoparticles for Fluorescent Nanocomposites by High-Pressure Magnetron Sputtering. Materials.

[150] Mukherji S., Bharti S., Shukla G., Mukherji S. (2019). Synthesis and characterization of size-and shape-controlled silver nanoparticles. Phys. Sci. Rev..

[151] Zhang R.-C., Sun D., Zhang R., Lin W.-F., Macias-Montero M., Patel J., Askari S., McDonald C., Mariotti D., Maguire P. (2017). Gold nanoparticle-polymer nanocomposites synthesized by room temperature atmospheric pressure plasma and their potential for fuel cell electrocatalytic application. Sci. Rep..

[152] Alamdari S., Sasani Ghamsari M., Lee C., Han W., Park H.-H., Tafreshi M.J., Afarideh H., Ara M.H.M. (2020). Preparation and characterization of zinc oxide nanoparticles using leaf extract of *Sambucus ebulus*. Appl. Sci..

[153] Ahmad A., Wei Y., Syed F., Tahir K., Rehman A.U., Khan A., Ullah S., Yuan Q. (2017). The effects of bacteria-nanoparticles interface on the antibacterial activity of green synthesized silver nanoparticles. Microb. Pathog..

[154] Esmail R., Afshar A., Morteza M., Abolfazl A., Akhondi E. (2022). Synthesis of silver nanoparticles with high efficiency and stability by culture supernatant of Bacillus ROM6 isolated from Zarshouran gold mine and evaluating its antibacterial effects. BMC Microbiol..

[155] Ibrahim S., Ahmad Z., Manzoor M.Z., Mujahid M., Faheem Z., Adnan A. (2021). Optimization for biogenic microbial synthesis of silver nanoparticles through response surface methodology, characterization, their antimicrobial, antioxidant, and catalytic potential. Sci. Rep..

[156] Kumar M., Upadhyay L.S., Kerketta A., Vasanth D. (2022). Extracellular Synthesis of Silver Nanoparticles Using a Novel Bacterial Strain Kocuria rhizophila BR-1: Process Optimization and Evaluation of Antibacterial Activity. BioNanoScience.

[157] Sharma S., Sharma N., Kaushal N. (2023). Utilization of novel bacteriocin synthesized silver nanoparticles (AgNPs) for their application in antimicrobial packaging for preservation of tomato fruit. Front. Sustain. Food Syst..

[158] Doman K.M., Gharieb M.M., Abd El-Monem A.M., Morsi H.H. (2024). Synthesis of silver and copper nanoparticle using Spirulina platensis and evaluation of their anticancer activity. Int. J. Environ. Health Res..

[159] Almatroudi A. (2020). Silver nanoparticles: Synthesis, characterisation and biomedical applications. Open Life Sci..

[160] Vijayaram S., Razafindralambo H., Sun Y.-Z., Vasantharaj S., Ghafarifarsani H., Hoseinifar S.H., Raeeszadeh M. (2024). Applications of Green Synthesized Metal Nanoparticles—A Review. Biol. Trace Elem. Res..

[161] Parveen M., Kumar A., Khan M.S., Rehman R., Furkan M., Khan R.H., Nami S.A. (2022). Comparative study of biogenically synthesized silver and gold nanoparticles of *Acacia auriculiformis* leaves and their efficacy against Alzheimer’s and Parkinson’s disease. Int. J. Biol. Macromol..

[162] Rathinavel S., Priyadharshini K., Panda D. (2021). A review on carbon nanotube: An overview of synthesis, properties, functionalization, characterization, and the application. Mater. Sci. Eng. B.

[163] Moteriya P., Chanda S. (2017). Synthesis and characterization of silver nanoparticles using Caesalpinia pulcherrima flower extract and assessment of their in vitro antimicrobial, antioxidant, cytotoxic, and genotoxic activities. Artif. Cells Nanomed. Biotechnol..

[164] Zhang X.-F., Liu Z.-G., Shen W., Gurunathan S. (2016). Silver nanoparticles: Synthesis, characterization, properties, applications, and therapeutic approaches. Int. J. Mol. Sci..

[165] Martí-Rujas J. (2020). Structural elucidation of microcrystalline MOFs from powder X-ray diffraction. Dalton Trans..

[166] Basher N.A., Abdulkhabeer A. (2022). Synthesis of novel demulsifier nano-materials and their application in the oil industry. Mater. Today Proc..

[167] Epp J. (2016). X-ray diffraction (XRD) techniques for materials characterization. Materials Characterization Using Nondestructive Evaluation (NDE) Methods.

[168] Ali A., Chiang Y.W., Santos R.M. (2022). X-ray diffraction techniques for mineral characterization: A review for engineers of the fundamentals, applications, and research directions. Minerals.

[169] Liu H., Hao C., Zhang Y., Yang H., Sun R. (2021). The interaction of graphene oxide-silver nanoparticles with trypsin: Insights from adsorption behaviors, conformational structure and enzymatic activity investigations. Colloids Surf. B Biointerfaces.

[170] Shaik M.R., Khan M., Kuniyil M., Al-Warthan A., Alkhathlan H.Z., Siddiqui M.R.H., Shaik J.P., Ahamed A., Mahmood A., Khan M. (2018). Plant-extract-assisted green synthesis of silver nanoparticles using *Origanum vulgare* L. extract and their microbicidal activities. Sustainability.

[171] Nath D., Singh F., Das R. (2020). X-ray diffraction analysis by Williamson-Hall, Halder-Wagner and size-strain plot methods of CdSe nanoparticles-a comparative study. Mater. Chem. Phys..

[172] Huang H., Jiang Q., Chen Y., Li X., Mao X., Chen X., Huang L., Gao W. (2016). Preparation, physico–chemical characterization and biological activities of two modified starches from yam (*Dioscorea Opposita* Thunb.). Food Hydrocoll..

[173] Yadav M., Baboo P., Gupta N., Arora V. (2018). Composition and Characterisation of Argent Nanoparticles and Argent Bionanocomposities. Asian J. Res. Chem..

[174] Sharma N.K., Vishwakarma J., Rai S., Alomar T.S., AlMasoud N., Bhattarai A. (2022). Green Route Synthesis and Characterization Techniques of Silver Nanoparticles and Their Biological Adeptness. ACS Omega.

[175] Verma P., Maheshwari S.K. (2018). Preparation of sliver and selenium nanoparticles and its characterization by dynamic light scattering and scanning electron microscopy. J. Microsc. Ultrastruct..

[176] Ghojavand S., Madani M., Karimi J. (2020). Green synthesis, characterization and antifungal activity of silver nanoparticles using stems and flowers of felty germander. J. Inorg. Organomet. Polym. Mater..

[177] Babick F. (2020). Dynamic light scattering (DLS). Characterization of Nanoparticles.

[178] Patil R.B., Chougale A.D. (2021). Analytical methods for the identification and characterization of silver nanoparticles: A brief review. Mater. Today Proc..

[179] Zhang J., Ma J., Zhang S., Wang W., Chen Z. (2015). A highly sensitive nonenzymatic glucose sensor based on CuO nanoparticles decorated carbon spheres. Sens. Actuators B Chem..

[180] Ma Y.-F., Wang L.-J., Zhou Y.-L., Zhang X.-X. (2019). A facilely synthesized glutathione-functionalized silver nanoparticle-grafted covalent organic framework for rapid and highly efficient enrichment of N-linked glycopeptides. Nanoscale.

[181] Peets P., Kaupmees K., Vahur S., Leito I. (2019). Reflectance FT-IR spectroscopy as a viable option for textile fiber identification. Herit. Sci..

[182] Cavezza F., Pletincx S., Revilla R.I., Weaytens J., Boehm M., Terryn H., Hauffman T. (2019). Probing the metal oxide/polymer molecular hybrid interfaces with nanoscale resolution using AFM-IR. J. Phys. Chem. C.

[183] Rodriguez-Saona L., Aykas D.P., Borba K.R., Urtubia A. (2020). Miniaturization of optical sensors and their potential for high-throughput screening of foods. Curr. Opin. Food Sci..

[184] Ridier K., Bas A.-C., Zhang Y., Routaboul L., Salmon L., Molnár G., Bergaud C., Bousseksou A. (2020). Unprecedented switching endurance affords for high-resolution surface temperature mapping using a spin-crossover film. Nat. Commun..

[185] Crucho C.I., Barros M.T. (2017). Polymeric nanoparticles: A study on the preparation variables and characterization methods. Mater. Sci. Eng. C.

[186] Khandel P., Shahi S.K. (2018). Mycogenic nanoparticles and their bio-prospective applications: Current status and future challenges. J. Nanostructure Chem..

[187] Ratnayake S., Lützenkirchen J., Finck N., Schild D., Heberling F., Gil-Díaz T., Dardenne K., Rothe J., Geckeis H. (2023). Combined X-ray absorption and SEM–EDX spectroscopic analysis for the speciation of thorium in soil. Sci. Rep..

[188] Vahedikia N., Garavand F., Tajeddin B., Cacciotti I., Jafari S.M., Omidi T., Zahedi Z. (2019). Biodegradable zein film composites reinforced with chitosan nanoparticles and cinnamon essential oil: Physical, mechanical, structural and antimicrobial attributes. Colloids Surf. B Biointerfaces.

[189] Parent L.R., Bakalis E., Ramírez-Hernández A., Kammeyer J.K., Park C., De Pablo J., Zerbetto F., Patterson J.P., Gianneschi N.C. (2017). Directly observing micelle fusion and growth in solution by liquid-cell transmission electron microscopy. J. Am. Chem. Soc..

[190] Mendes R.G., Pang J., Bachmatiuk A., Ta H.Q., Zhao L., Gemming T., Fu L., Liu Z., Rummeli M.H. (2019). Electron-driven in situ transmission electron microscopy of 2D transition metal dichalcogenides and their 2D heterostructures. ACS Nano.

[191] Pishgar S., Gulati S., Strain J.M., Liang Y., Mulvehill M.C., Spurgeon J.M. (2021). In situ analytical techniques for the investigation of material stability and interface dynamics in electrocatalytic and photoelectrochemical applications. Small Methods.

[192] Yin W., Brittain D., Borseth J., Scott M.E., Williams D., Perkins J., Own C.S., Murfitt M., Torres R.M., Kapner D. (2020). A petascale automated imaging pipeline for mapping neuronal circuits with high-throughput transmission electron microscopy. Nat. Commun..

[193] Ullah H., Lun L., Riaz L., Naseem F., Shahab A., Rashid A. (2021). Physicochemical characteristics and thermal degradation behavior of dry and wet torrefied orange peel obtained by dry/wet torrefaction. Biomass Convers. Biorefinery.

[194] Akash M.S.H., Rehman K., Akash M.S.H., Rehman K. (2020). Thermo gravimetric analysis. Essent. Pharm. Anal..

[195] Lin S.-Y. (2016). An overview of advanced hyphenated techniques for simultaneous analysis and characterization of polymeric materials. Crit. Rev. Solid State Mater. Sci..

[196] Manivasagan P., Kang K.-H., Kim D.G., Kim S.-K. (2015). Production of polysaccharide-based bioflocculant for the synthesis of silver nanoparticles by *Streptomyces* sp.. Int. J. Biol. Macromol..

[197] Nabil-Adam A., Shreadah M.A. (2020). Biogenic silver nanoparticles synthesis from new record aquatic bacteria of *Nile tilapia* and evaluation of their biological activity. J. Pure Appl. Microbiol..

[198] Yumei L., Yamei L., Qiang L., Jie B. (2017). Rapid biosynthesis of silver nanoparticles based on flocculation and reduction of an exopolysaccharide from *Arthrobacter* sp. B4: Its antimicrobial activity and phytotoxicity. J. Nanomater..

[199] Barkat M.A., Beg S., Naim M., Pottoo F.H., Singh S.P., Ahmad F.J. (2018). Current progress in synthesis, characterization and applications of silver nanoparticles: Precepts and prospects. Recent Pat. Anti-Infect. Drug Discov..

[200] Naganthran A., Verasoundarapandian G., Khalid F.E., Masarudin M.J., Zulkharnain A., Nawawi N.M., Karim M., Che Abdullah C.A., Ahmad S.A. (2022). Synthesis, characterization and biomedical application of silver nanoparticles. Materials.

[201] Patave T., Siddiqui A.R. (2018). Silver nanoparticles: Green synthesis, characterization and its applications. Asian J. Pharm. Pharmacol..

[202] Ahmed S., Ahmad M., Swami B.L., Ikram S. (2016). A review on plants extract mediated synthesis of silver nanoparticles for antimicrobial applications: A green expertise. J. Adv. Res..

[203] Ali H.R., Emam A.N., Hefny E.G., Koraney N.F., Mansour A.S., Salama A.M., Ali S.F., Aboolo S.H., Shahein M.A. (2021). Silver nanoparticles enhance the effectiveness of traditional antibiotics against *S. aureus* causing bovine mastitis within the safety limit. J. Nanoparticle Res..

[204] Zharkova M.S., Orlov D.S., Golubeva O.Y., Chakchir O.B., Eliseev I.E., Grinchuk T.M., Shamova O.V. (2019). Application of antimicrobial peptides of the innate immune system in combination with conventional antibiotics—A novel way to combat antibiotic resistance?. Front. Cell. Infect. Microbiol..

[205] Ghiuță I., Cristea D., Croitoru C., Kost J., Wenkert R., Vyrides I., Anayiotos A., Munteanu D. (2018). Characterization and antimicrobial activity of silver nanoparticles, biosynthesized using Bacillus species. Appl. Surf. Sci..

[206] Natarajan K. (2017). Use of bioflocculants for mining environmental control. Trans. Indian Inst. Met..

[207] Vazquez-Muñoz R., Meza-Villezcas A., Fournier P., Soria-Castro E., Juarez-Moreno K., Gallego-Hernández A., Bogdanchikova N., Vazquez-Duhalt R., Huerta-Saquero A. (2019). Enhancement of antibiotics antimicrobial activity due to the silver nanoparticles impact on the cell membrane. PLoS ONE.

[208] Naidu K., Adam J., Govender P. (2015). Biomedical applications and toxicity of nanosilver: A review. Med. Technol. SA.

[209] Tripathi N., Goshisht M.K. (2022). Recent advances and mechanistic insights into antibacterial activity, antibiofilm activity, and cytotoxicity of silver nanoparticles. ACS Appl. Bio Mater..

[210] Gudikandula K., Charya Maringanti S. (2016). Synthesis of silver nanoparticles by chemical and biological methods and their antimicrobial properties. J. Exp. Nanosci..

[211] Verma P., Maheshwari S.K. (2019). Applications of Silver nanoparticles in diverse sectors. Int. J. Nano Dimens..

[212] Tang S., Zheng J. (2018). Antibacterial activity of silver nanoparticles: Structural effects. Adv. Healthc. Mater..

[213] Hwang S., Han C.W., Venkatakrishnan S.V., Bouman C.A., Ortalan V. (2017). Towards the low-dose characterization of beam sensitive nanostructures via implementation of sparse image acquisition in scanning transmission electron microscopy. Meas. Sci. Technol..

[214] Saravanan M., Barik S.K., MubarakAli D., Prakash P., Pugazhendhi A. (2018). Synthesis of silver nanoparticles from *Bacillus brevis* (NCIM 2533) and their antibacterial activity against pathogenic bacteria. Microb. Pathog..

[215] Mallmann E.J., Cunha F.A., Castro B.N., Maciel A.M., Menezes E.A., Fechine P.B. (2015). Antifungal activity of silver nanoparticles obtained by green synthesis. Rev. Inst. Med. Trop. Sao Paulo.

[216] Khatoon N., Mishra A., Alam H., Manzoor N., Sardar M. (2015). Biosynthesis, characterization, and antifungal activity of the silver nanoparticles against pathogenic *Candida* species. BioNanoScience.

[217] Rawla P., Sunkara T., Barsouk A. (2019). Epidemiology of colorectal cancer: Incidence, mortality, survival, and risk factors. Gastroenterol. Rev./Przegląd Gastroenterol..

[218] Patra J.K., Das G., Fraceto L.F., Campos E.V.R., Rodriguez-Torres M.d.P., Acosta-Torres L.S., Diaz-Torres L.A., Grillo R., Swamy M.K., Sharma S. (2018). Nano based drug delivery systems: Recent developments and future prospects. J. Nanobiotechnol..

[219] Ibrahim E.H., Ghramh H.A., Alshehri A., Kilany M., Khalofah A., El-Mekkawy H.I., Sayed M.A., Alothaid H., Taha R. (2021). *Lepidium sativum* and its biogenic silver nanoparticles activate immune cells and induce apoptosis and cell cycle arrest in HT-29 colon cancer cells. J. Biomater. Tissue Eng..

[220] Prema P., Veeramanikandan V., Rameshkumar K., Gatasheh M.K., Hatamleh A.A., Balasubramani R., Balaji P. (2022). Statistical optimization of silver nanoparticle synthesis by green tea extract and its efficacy on colorimetric detection of mercury from industrial waste water. Environ. Res..

[221] Bhatt P., Pandey S.C., Joshi S., Chaudhary P., Pathak V.M., Huang Y., Wu X., Zhou Z., Chen S. (2022). Nanobioremediation: A sustainable approach for the removal of toxic pollutants from the environment. J. Hazard. Mater..

[222] Sudarman F., Shiddiq M., Armynah B., Tahir D. (2023). Silver nanoparticles (AgNPs) synthesis methods as heavy-metal sensors: A review. Int. J. Environ. Sci. Technol..

[223] Zahran M., Khalifa Z., Zahran M.A.-H., Azzem M.A. (2021). Recent advances in silver nanoparticle-based electrochemical sensors for determining organic pollutants in water: A review. Mater. Adv..

[224] Bhardwaj A.K., Sundaram S., Yadav K.K., Srivastav A.L. (2021). An overview of silver nano-particles as promising materials for water disinfection. Environ. Technol. Innov..

[225] Gadkari R.R., Ali S.W., Alagirusamy R., Das A. (2018). Silver nanoparticles in water purification: Opportunities and challenges. Mod. Age Environ. Probl. Their Remediat..

[226] Liu H., Lai W., Liu X., Yang H., Fang Y., Tian L., Li K., Nie H., Zhang W., Shi Y. (2021). Exposure to copper oxide nanoparticles triggers oxidative stress and endoplasmic reticulum (ER)-stress induced toxicology and apoptosis in male rat liver and BRL-3A cell. J. Hazard. Mater..

[227] Mohammed A.N. (2019). Resistance of bacterial pathogens to calcium hypochlorite disinfectant and evaluation of the usability of treated filter paper impregnated with nanosilver composite for drinking water purification. J. Glob. Antimicrob. Resist..

[228] Allam N.G., Ismail G.A., El-Gemizy W.M., Salem M.A. (2019). Biosynthesis of silver nanoparticles by cell-free extracts from some bacteria species for dye removal from wastewater. Biotechnol. Lett..

[229] Krishna G., Pranitha V., Sundaram R., Charya M.S. (2020). Biogenic Synthesis of Silver Nanoparticles and Their Applications. Functionalized Nanomaterials I.

[230] Olas B., Białecki J., Urbańska K., Bryś M. (2021). The effects of natural and synthetic blue dyes on human health: A review of current knowledge and therapeutic perspectives. Adv. Nutr..

[231] Gola D., Bhatt N., Bajpai M., Singh A., Arya A., Chauhan N., Srivastava S.K., Tyagi P.K., Agrawal Y. (2021). Silver nanoparticles for enhanced dye degradation. Curr. Res. Green Sustain. Chem..

[232] Marimuthu S., Antonisamy A.J., Malayandi S., Rajendran K., Tsai P.-C., Pugazhendhi A., Ponnusamy V.K. (2020). Silver nanoparticles in dye effluent treatment: A review on synthesis, treatment methods, mechanisms, photocatalytic degradation, toxic effects and mitigation of toxicity. J. Photochem. Photobiol. B Biol..

[233] Sana S.S., Haldhar R., Parameswaranpillai J., Chavali M., Kim S.-C. (2022). Silver nanoparticles-based composite for dye removal: A comprehensive review. Clean. Mater..

[234] Ali M.H., Goher M.E., Al-Afify A.D., El-Sayed S.M. (2022). A facile method for synthesis rGO/Ag nanocomposite and its uses for enhancing photocatalytic degradation of Congo red dye. SN Appl. Sci..

[235] Anil A., Sanjeev K., Kamarudheen N., Sebastian P.M., Rao K.B. (2023). EPS-mediated biosynthesis of nanoparticles by *Bacillus stratosphericus* A07, their characterization and potential application in azo dye degradation. Arch. Microbiol..

[236] Jain K., Kumar Mehra N., Jain N.K. (2015). Nanotechnology in drug delivery: Safety and toxicity issues. Curr. Pharm. Des..

[237] Saravana Kumar P., Balachandran C., Duraipandiyan V., Ramasamy D., Ignacimuthu S., Al-Dhabi N.A. (2015). Extracellular biosynthesis of silver nanoparticle using *Streptomyces* sp. 09 PBT 005 and its antibacterial and cytotoxic properties. Appl. Nanosci..

[238] Karuppaiya P., Satheeshkumar E., Tsay H.S. (2019). Biogenic synthesis of silver nanoparticles using rhizome extract of Dysosma pleiantha and its antiproliferative effect against breast and human gastric cancer cells. Mol. Biol. Rep..

[239] Dey Bhowmik A., Bandyopadhyay A., Chattopadhyay A. (2019). Cytotoxic and mutagenic effects of green silver nanoparticles in cancer and normal cells: A brief review. Nucleus.

[240] Choudhary A., Singh S., Ravichandiran V. (2022). Toxicity, preparation methods and applications of silver nanoparticles: An update. Toxicol. Mech. Methods.

[241] Eghbalifam N., Shojaosadati S.A., Hashemi-Najafabadi S., Khorasani A.C. (2020). Synthesis and characterization of antimicrobial wound dressing material based on silver nanoparticles loaded gum Arabic nanofibers. Int. J. Biol. Macromol..

